# Targeting NXPH4/ALDH1L2 signaling suppresses enzalutamide resistance in prostate cancer

**DOI:** 10.1038/s41420-026-02944-z

**Published:** 2026-02-04

**Authors:** Xianchao Sun, Ying Zhang, Wei Zhang, Liang Jin, Shiyong Xin

**Affiliations:** 1 Department of Urology, the Second Affiliated Hospital of Anhui Medical University, Hefei, China; 2 Department of Urology, the First Affiliated Hospital of Wannan Medical College (Yijishan Hospital of Wannan Medical College), Wuhu, China; 3 Department of Urology, the Second Affiliated Hospital, Zhejiang University School of Medicine, Hangzhou, China; 4 Department of Urology, the First Affiliated Hospital and College of Clinical Medicine of Henan University of Science and Technology, Luoyang, China

**Keywords:** Prostate cancer, Drug development

## Abstract

While androgen receptor (AR) pathway inhibitors such as enzalutamide have demonstrated significant therapeutic efficacy in prostate cancer (PCa) treatment, the inevitable development of acquired resistance continues to pose a major clinical challenge in managing advanced PCa. We characterized Neurexophilin 4 (NXPH4) as a contributor to enzalutamide resistance (EnzR). Gain- and loss-of-function studies were conducted in PCa cell lines and mouse subcutaneous xenograft models to elucidate the role of NXPH4 in castration-resistant prostate cancer (CRPC). Additionally, the regulatory mechanisms of gene expression were assessed using a series of molecular and biochemical experiments. Our study demonstrates that AR as a transcriptional activator of NXPH4. Elevated NXPH4 expression facilitated PCa proliferation under enzalutamide treatment through mitochondrial metabolic reprogramming. We identified that NXPH4 partially localizes to mitochondria and physically interacts with aldehyde dehydrogenase 1 family member L2 (ALDH1L2), a critical enzyme in one-carbon metabolism. Androgen deprivation stimulated NXPH4 mitochondrial translocation and enhanced its binding to ALDH1L2. NXPH4-mediated metabolic reprogramming promotes PCa progression. Notably, the combination of NXPH4 knockdown and enzalutamide treatment showed potent synergistic effects, significantly suppressing cell proliferation in vitro and substantially inhibiting tumor growth in vivo. These findings reveal a previously unrecognized mechanism of EnzR and identify the NXPH4-ALDH1L2 complex as a promising therapeutic target for CRPC treatment.

## Introduction

Prostate cancer (PCa) is one of the most common malignancies of the male reproductive system, with rapidly increasing incidence and mortality rates worldwide [1, 2]. Accurate diagnosis and effective treatment of PCa remain critical medical challenges that urgently need to be addressed [3]. The progression of PCa is closely linked to the androgen receptor (AR). The binding of androgens to AR triggers the expression of AR-regulated genes, thereby driving PCa growth. As a result, androgen deprivation therapy (ADT), which aims to suppress androgen production or block AR signaling through surgical or pharmacological castration, is a cornerstone of PCa treatment [4]. While most patients initially respond well to ADT, they inevitably develop castration-resistant prostate cancer (CRPC) [5]. Enzalutamide, a next-generation androgen receptor pathway inhibitor, is currently a primary treatment option for CRPC. However, despite its initial efficacy, patients often develop resistance within a short period, leaving a significant unmet clinical need for effective therapeutic strategies [6].

Multiple molecular mechanisms underlie the improved prognosis observed in CRPC patients [7]. However, the development of drug resistance remains a significant therapeutic challenge in CRPC management. Resistance to enzalutamide arises from the activation and interplay of multiple complex signaling pathways. Enzalutamide resistance (EnzR) is broadly categorized into AR-dependent and AR-independent mechanisms. AR-dependent resistance mechanisms include AR amplification and overexpression, AR mutations, the generation of AR splice variants, and increased synthesis of dihydrotestosterone (DHT) [7–9]. In contrast, AR-independent resistance mechanisms involve glucocorticoid receptor overexpression and upregulation, which restore the expression of certain AR target genes, as well as epithelial-mesenchymal transition, neuroendocrine differentiation and immune system activation [10–12]. Given the lack of effective treatments for CRPC patients who develop enzalutamide resistance, it is critical to elucidate the underlying mechanisms of resistance and identify strategies to overcome it. To uncover potential driver genes of enzalutamide resistance in PCa, we conducted analyses and identified Neurexophilin 4 (NXPH4) as a candidate enzalutamide-resistant driver gene in PCa for further validation.

NXPH4, a member of the neurexophilin family, has been implicated in the pathogenesis of multiple cancer types and promotes tumor initiation and progression [13–15]. Notably, NXPH4 has also been associated with chemoresistance in several malignancies. However, the functional role of NXPH4 in PCa, particularly in CRPC, remains poorly understood. In bladder cancer, NXPH4 has been shown to enhance glycolysis, with its overexpression leading to increased glycolytic activity [14]. Similarly, in colorectal cancer, NXPH4 as a novel prognostic biomarker and promotes progression [13]. Our findings demonstrate that NXPH4 expression is significantly upregulated in EnzR cell lines and clinical specimens. Functional studies reveal that NXPH4 promotes PCa progression, particularly facilitating the transition to CRPC. Previous studies have shown that NXPH4 is involved in maintaining redox homeostasis and metabolic adaptation. Enzalutamide-resistant PCa cells are known to undergo extensive metabolic reprogramming, especially in mitochondrial function and oxidative stress regulation. This suggested to us that NXPH4 may play a similar adaptive role in PCa under enzalutamide pressure. Intriguingly, we observed mitochondrial localization of NXPH4, where it physically interacts with aldehyde dehydrogenase 1 family member L2 (ALDH1L2) under androgen-deprived conditions. As the primary enzyme governing mitochondrial NADPH generation, ALDH1L2 plays a critical role in redox homeostasis, with its deficiency leading to profound mitochondrial dysfunction and oxidative stress susceptibility [16, 17]. NXPH4 regulates ALDH1L2-mediated mitochondrial function to promote CRPC progression.

In this study, we provide novel evidence highlighting the pivotal role of NXPH4 in regulating enzalutamide resistance in PCa. Specifically, we demonstrate that NXPH4 drives enzalutamide resistance by activating ALDH1L2 and reprogramming mitochondrial metabolism in PCa cells.

## Results

### Elevated expression of NXPH4 is significantly associated with enzalutamide resistance in PCa cells

**Fig. 1:**
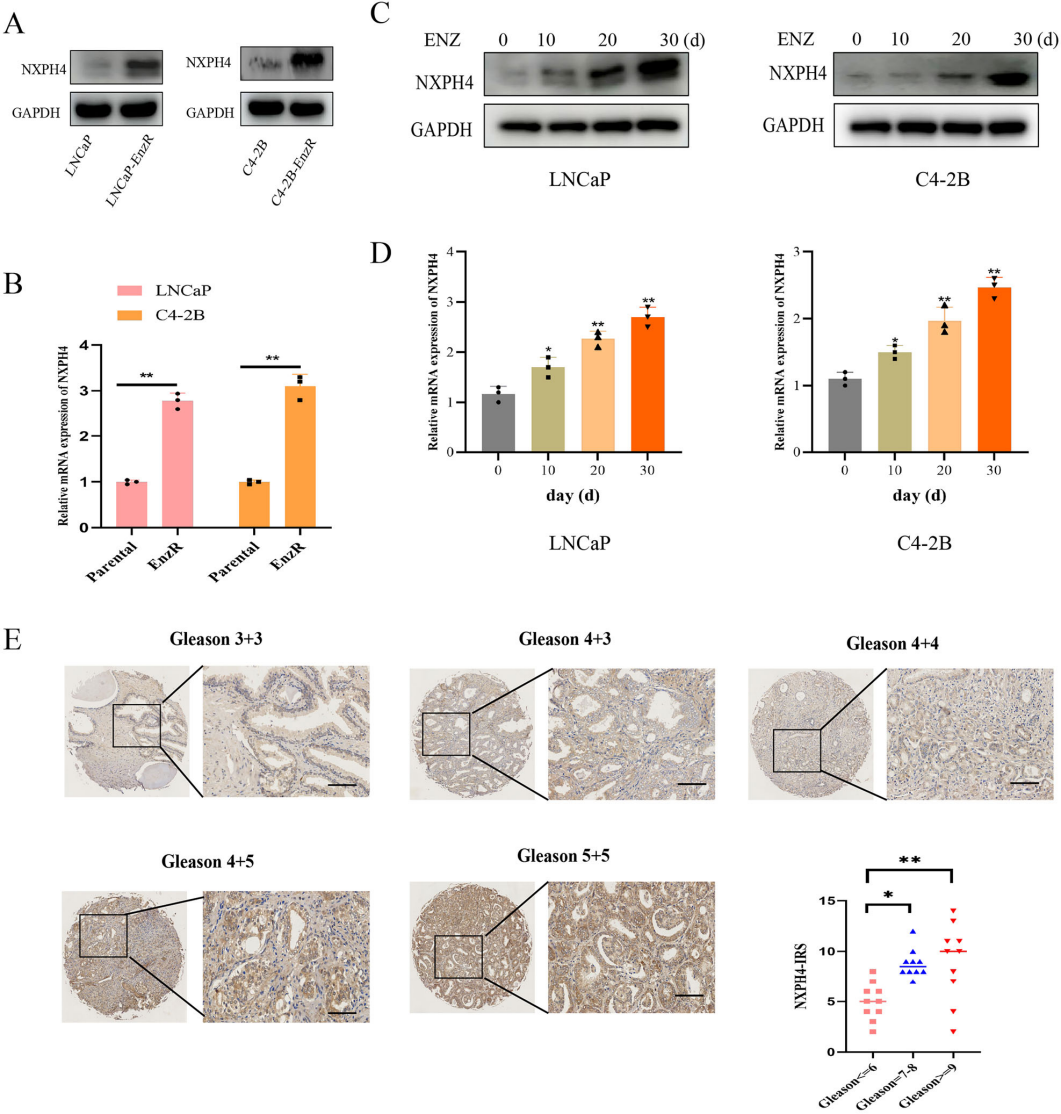
Elevated expression of NXPH4 is significantly associated with enzalutamide resistance in PCa cells. The expression of NXPH4 in enzalutamide sensitive and resistant LNCaP and C4-2B cells. RT-qPCR and western blotting assays were used to assess protein level (**A**) and mRNA level (**B**), respectively. The protein level and mRNA level of NXPH4 during prolonged enzalutamide treatment of LNCaP and C4-2B cells. LNCaP and C4-2B cells were treated with 1 µM enzalutamide for 0–30 days. NXPH4 protein (**C**) and mRNA (**D**) levels were detected at the indicated time points by western blotting and RT-qPCR. **E** IHC analysis of NXPH4 expression in PCa tissues with different Gleason scores. Scale bar, 50 µm. ENZ Enzalutamide. EnzR Enzalutamide Resistance. *P < 0.05, **P < 0.01.

To investigate the mechanism of enzalutamide resistance in PCa, we constructed two resistant cell model (LNCaP-EnzR and C4-2B-EnzR). The results of CCK-8, flow cytometry analysis and plate cloning experiments indicated that the cell proliferation and clone formation ability of EnzR cells were significantly enhanced compared to the control group (Supplementary Fig. 1). We utilized qRT-PCR and western blotting assays to detect NXPH4 expression levels in EnzR cells and demonstrated that the mRNA and protein expression levels of NXPH4 were significantly increased in EnzR cells relative to parental cells (Fig. 1A, B). Additionally, prolonged enzalutamide treatment (0–30 days) in PCa cells resulted in a significant upregulation of NXPH4 expression (Fig. 1C, D). Furthermore, IHC analysis revealed a positive correlation between NXPH4 expression levels and Gleason scores (Fig. 1E). To further validate the association between NXPH4 expression and enzalutamide resistance in clinical samples, IHC staining was performed in enzalutamide-sensitive and -resistant PCa specimens. The results showed that NXPH4 expression was markedly higher in enzalutamide-resistant tissues compared with enzalutamide-sensitive specimens (Supplementary Fig. 2A). These findings demonstrate that NXPH4 expression is markedly elevated in enzalutamide-resistant PCa.

### NXPH4 promotes EnzR and drives the progression of EnzR tumors

**Fig. 2:**
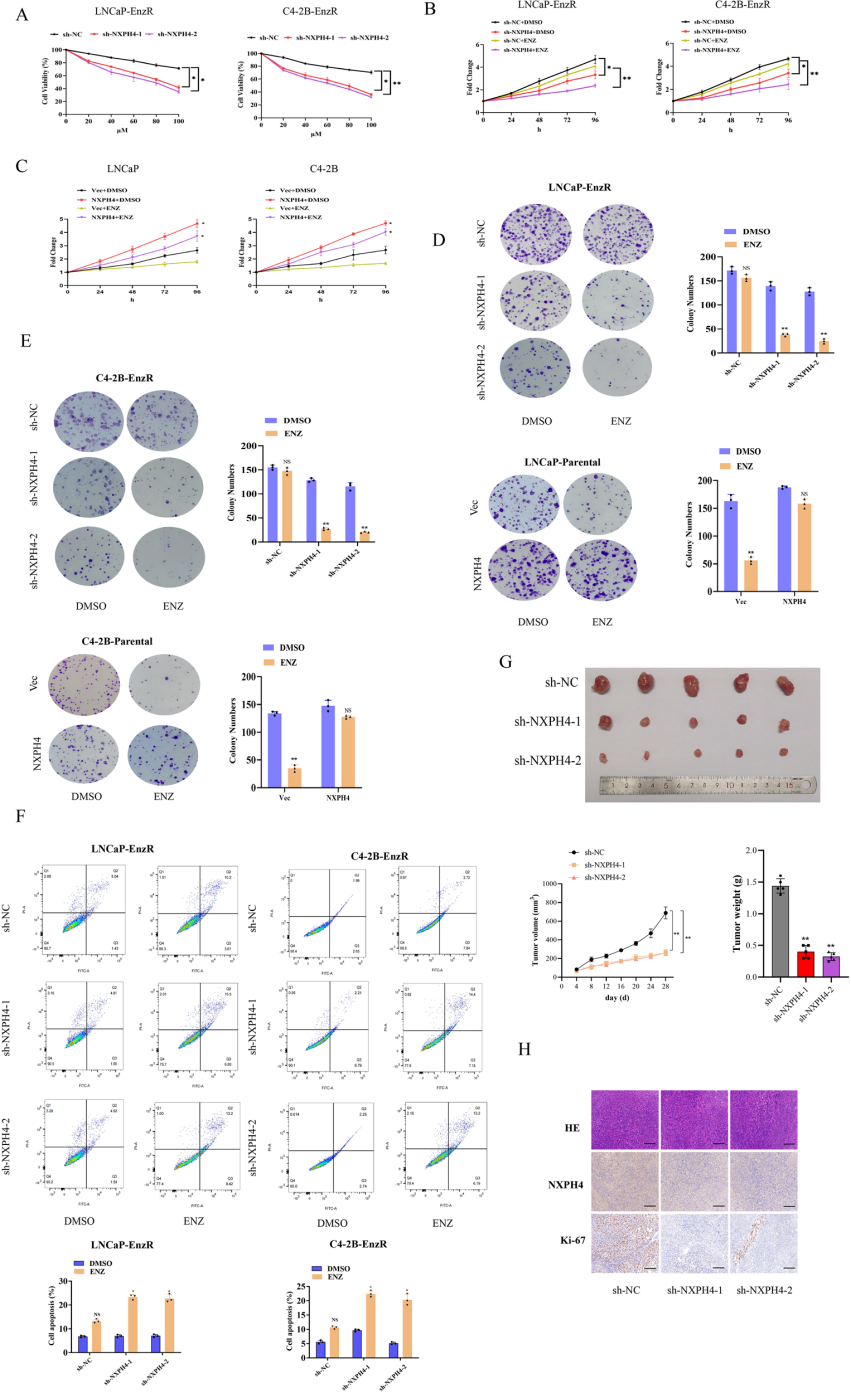
NXPH4 promotes EnzR and drives the progression of EnzR tumors. **A** Cell viability measured by CCK-8 assay in the indicated cell lines under enzalutamide treatment. **B**–**E** Cell proliferation assessed by CCK-8 assay and colony formation assay in indicated cells. LNCaP-ENZR and C4-2B-ENZR cells were transfected with sh-NC or NXPH4 shRNA (sh-NXPH4, stable transfection) and LNCaP and C4-2B cells were transfected with empty vector (Vec) or NXPH4 overexpression plasmid. **F** Cell apoptosis was measured in the indicated cell lines under enzalutamide treatment. **G** C4-2B-EnzR cells with stable knockdown of NXPH4, as well as their parental controls, were subcutaneously injected into nude mice (*n* = 5/group). The tumor volume was measured every two days after nude mice were castrated. Tumors were isolated from mice, photographed, and weighed at the endpoint. **H** Representative images of HE and IHC staining of NXPH4 and Ki-67 in tumor sections isolated from different groups. Scale bar, 50 µm. ENZ: Enzalutamide. EnzR: Enzalutamide Resistance. *P < 0.05, **P < 0.01.

To elucidate the role of NXPH4 in the development of EnzR, we performed NXPH4 knockdown in EnzR cells and NXPH4 overexpression in parental cells. NXPH4 knockdown and overexpression efficiencies were assessed by Western blot analysis (Supplementary Fig. 2B and C). Depletion of NXPH4 in EnzR cells significantly enhanced the inhibitory effect of enzalutamide on cell growth following treatment with enzalutamide (Fig. 2A, B). Conversely, NXPH4 overexpression in parental cells attenuated the growth inhibition induced by enzalutamide. Furthermore, enzalutamide sensitivity was reduced in parental cells overexpressing NXPH4 and increased in EnzR cells with NXPH4 knockdown (Fig. 2C–E, Supplementary Fig. 2D). Flow cytometry analysis revealed that NXPH4 knockdown in EnzR cells led to a moderate increase in apoptosis (Fig. 2F). Additionally, inhibition of NXPH4 in C4-2B-EnzR xenografts significantly suppressed tumor proliferation. To investigate the role of NXPH4 in EnzR in vivo, we established an animal model of enzalutamide-resistant PCa. C4-2B-EnzR cells with stable NXPH4 knockdown (sh-NXPH4) and control cells (sh-NC) were subcutaneously injected into nude mice when tumor volumes reached approximately 350 mm³. NXPH4 knockdown decreased tumor growth speed and overall tumor weight (Fig. 2G). IHC staining revealed a marked decrease in the positive rate of the proliferation marker Ki-67 in the NXPH4-silenced group (Fig. 2H). These findings collectively indicate that NXPH4 promotes the proliferation and tumorigenicity of EnzR cells. Together, the data suggest that NXPH4 confers a survival advantage to PCa cells following enzalutamide treatment, thereby driving EnzR tumor growth.

### The AR positively regulates NXPH4 gene transcription

**Fig. 3:**
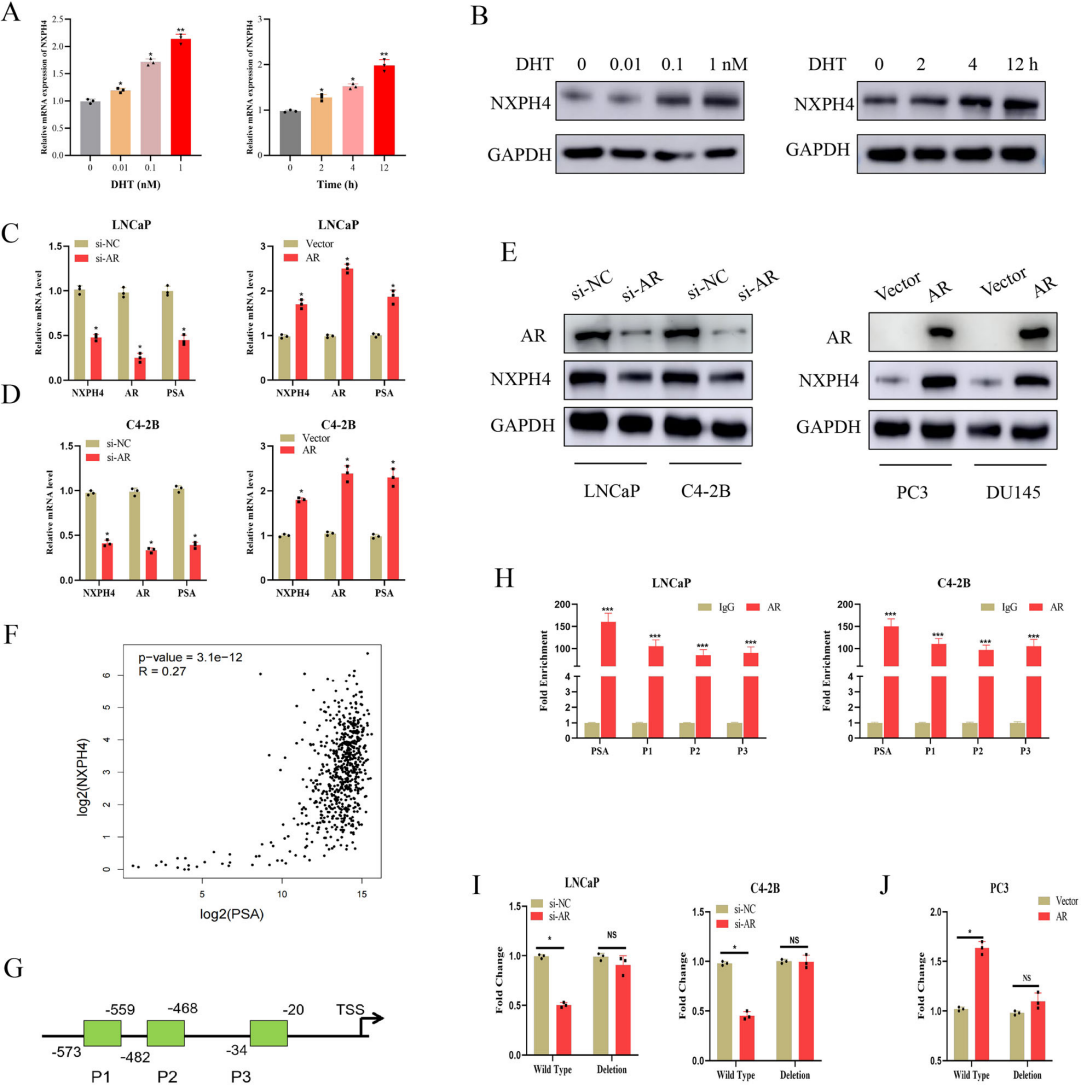
The AR positively regulates NXPH4 gene transcription. **A**, **B** LNCaP cells were cultured in CS-FBS medium for 4 days before challenged with 0–10 nM DHT for 24 h or 1 nM DHT for 0–48 h. The mRNA and protein expression levels of NXPH4 were measured by RT-qPCR and western blotting assays. **C**–**E** RT-qPCR and western blotting analysis of AR, NXPH4 and PSA in PCa cells that were treated with AR siRNA or AR expression plasmid. siNC, small interfering; NC, Negative control; Vec, vector. **F** Expression matrices (TPM) were log2-transformed (log2(TPM + 1)) and Pearson correlation between NXPH4 and PSA was calculated from TCGA cohort. **G** Schematic of three putative AR binding sites on the NXPH4 gene promoter. NXPH4 promoter was scanned for transcription factor motifs using JASPAR. Three amplicons (P1–P3) covering predicted regulatory regions were designed. According to motif analysis in JASPAR database, primers were designed as P1 (−20 to −34), P2 (−468 to −482) and P3 (−559 to −573) based on their distances to the transcription start site (TSS) of NXPH4 gene. **H** ChIP analysis was conducted in LNCaP and C4-2B cells to validate AR enrichment on NXPH4 promoter. Purified rabbit IgG was used as negative control. Primers fanking the AR binding site on the PSA gene promoter were used as positive controls. **I** Luciferase reporter assays were performed in LNCaP or C4-2B cells transfected with negative control (si-NC) or AR siRNA (si-AR) or PC3 cells transfected with empty vector **J**. *P < 0.05, **P < 0.01, ***P < 0.001.

To investigate whether NXPH4 is an androgen-regulated gene, we first assessed the impact of DHT treatment on NXPH4 expression. Our findings revealed a significant increase in both NXPH4 mRNA and protein levels following DHT treatment (Fig. 3A, B). Additionally, exogenous expression of the AR in PC3 and DU145 cells led to elevated NXPH4 expression, whereas AR silencing in LNCaP and C4-2B cells resulted in reduced NXPH4 levels (Fig. 3C–E). To further explore the regulatory relationship between AR and NXPH4, we analyzed the correlation between NXPH4 and the AR target gene PSA. As illustrated in Fig. 3F, NXPH4 mRNA expression in PCa samples from clinical cohorts in public datasets showed a positive correlation with PSA mRNA expression. Moreover, motif analysis identified three potential AR-binding sites within the NXPH4 promoter region, designated as P1 (−20 to −34), P2 (−468 to −482), and P3 (−559 to −573) (Fig. 3G). Site-specific chromatin immunoprecipitation (ChIP) assays confirmed significant enrichment of AR binding at these three sites in the NXPH4 promoter of LNCaP and C4-2B cells (Fig. 3H). Luciferase reporter assays demonstrated that exogenous AR expression in PC3 cells enhanced the activity of the wild-type NXPH4 promoter but not the mutant promoter. Conversely, AR knockdown reduced wild-type NXPH4 promoter activity in LNCaP and C4-2B cells (Fig. 3I, J). Taken together, these results provide strong evidence that AR functions as a transcriptional activator of NXPH4.

### NXPH4 can enhance mitochondrial function

**Fig. 4:**
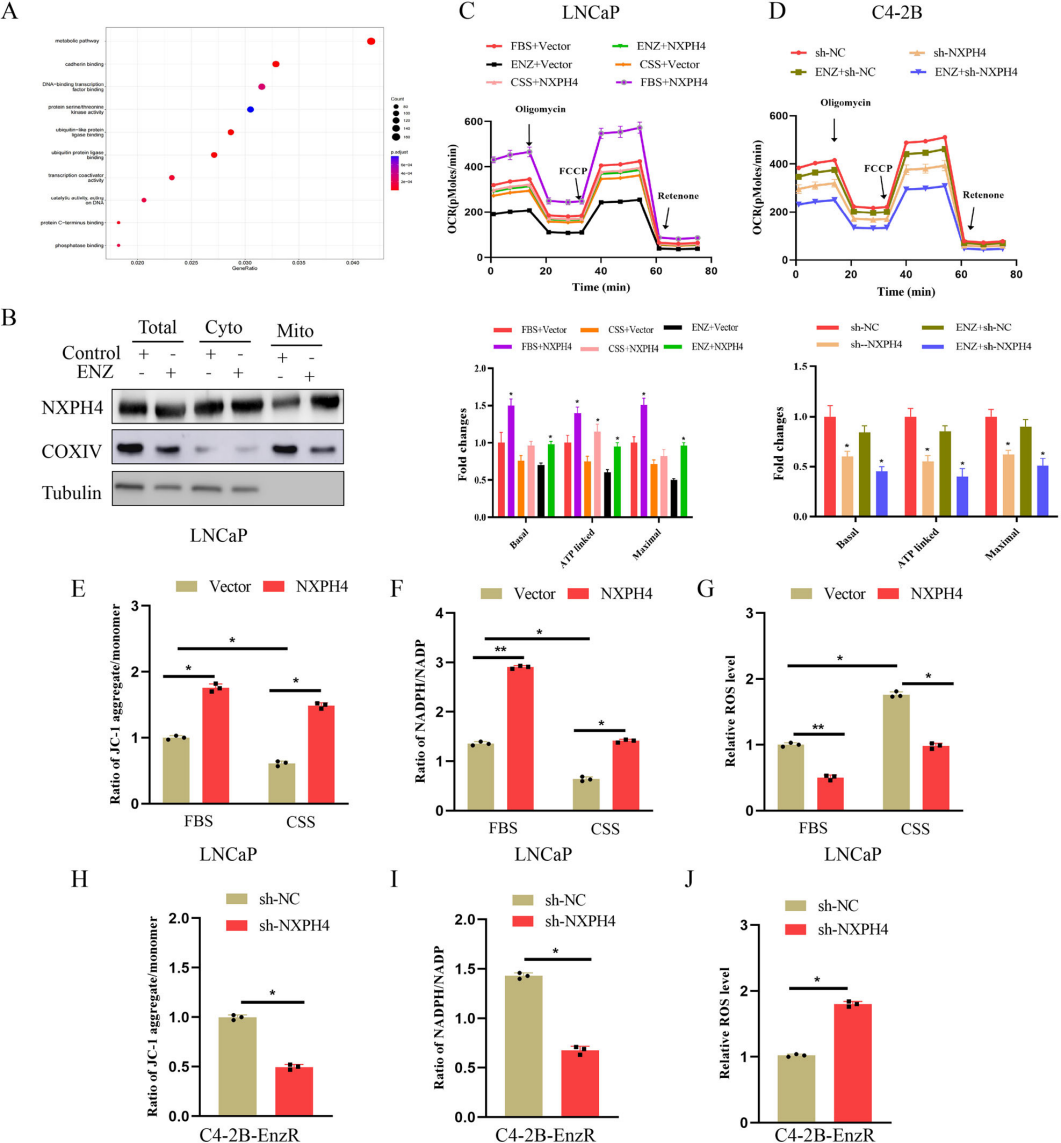
NXPH4 can enhance mitochondrial function. **A** KEGG pathway gene set enrichment analysis of downregulated genes in C4-2B cells with NXPH4 knockdown. **B** The protein levels of NXPH4 in total cell lysates (Total), cytosolic fraction (Cyto), and mitochondrial fraction (Mito) were analyzed by western blotting in LNCaP cells with or without androgen deprivation. COXIV and tubulin were used as mitochondrial and cytosolic markers. **C**, **D** Measurement of OCR in indicated cells with NXPH4 overexpression or knockdown and treated with or without androgen deprivation and enzalutamide (10 µM). Representative recording of OCR during extracellular flow Seahorse analysis is shown in the up panel, and quantitative analysis of the calculated basal and maximum respiratory rates, ATP production rate and spare respiratory capacity are shown in the bottom panel. **E**–**J** Measurement of membrane potentials, NADPH/ NADP^+^ ratio, and ROS levels in LNCaP and C4-2B cells with NXPH4 overexpression or knockdown and treated with or without androgen deprivation. FBS Fatal Bovine Serum. CSS Charcoal-Stripped Serum. ENZ Enzalutamide. EnzR Enzalutamide Resistance. *P < 0.05, **P < 0.01.

To elucidate the molecular mechanism through which NXPH4 promotes the aggressive phenotype of PCa cells, we conducted RNA sequencing in C4-2B cells with or without NXPH4 expression. Subsequent Kyoto Encyclopedia of Genes and Genomes (KEGG) pathway analysis revealed that the dysregulated genes resulting from NXPH4 knockdown were predominantly enriched in cellular metabolism-related pathways (Fig. 4A, Supplementary Table S3). Specifically, we examined the differential expression of genes involved in mitochondrial metabolism, including the tricarboxylic acid (TCA) cycle, electron transport chain (ETC), and redox balance pathways. The results revealed significant enrichment of genes associated with mitochondrial energy metabolism and oxidative stress regulation upon NXPH4 knockdown (Supplementary Fig. 3A). Further investigation using western blotting demonstrated that, in addition to its presence in the cytoplasmic compartment, a fraction of NXPH4 was localized to the mitochondria in LNCaP cells. Notably, mitochondrial NXPH4 expression increased in LNCaP cells upon treatment with enzalutamide (Fig. 4B). These findings suggest that a subset of NXPH4 localizes to the mitochondria in response to ADT and may play a role in regulating mitochondrial function. To investigate the effects of NXPH4 on mitochondrial function, we assessed mitochondrial respiration in PCa cells. As illustrated in Fig. 4C, overexpression of NXPH4 significantly reversed the decline in mitochondrial oxygen consumption rate (OCR) in LNCaP cells treated with enzalutamide, whereas NXPH4 knockdown exacerbated the reduction of OCR in C4-2B cells. We further evaluated the impact of NXPH4 on mitochondrial function by measuring mitochondrial membrane potential, the NADPH/NADP^+^ ratio, and reactive oxygen species (ROS) production. Androgen deprivation resulted in decreased mitochondrial membrane potential and NADPH/NADP^+^ ratio, along with increased mitochondrial ROS production in LNCaP cells. However, NXPH4 overexpression mitigated these mitochondrial dysfunctions induced by androgen deprivation (Fig. 4E–G, Supplementary Fig. 3B). Conversely, in NXPH4-depleted C4-2B cells, mitochondrial membrane potential and the NADPH/NADP^+^ ratio were reduced, while mitochondrial ROS production was elevated (Fig. 4H–J). Collectively, these findings demonstrate that NXPH4 enhances mitochondrial activity.

### NXPH4 enhances the mitochondrial function via ALDH1L2

**Fig. 5:**
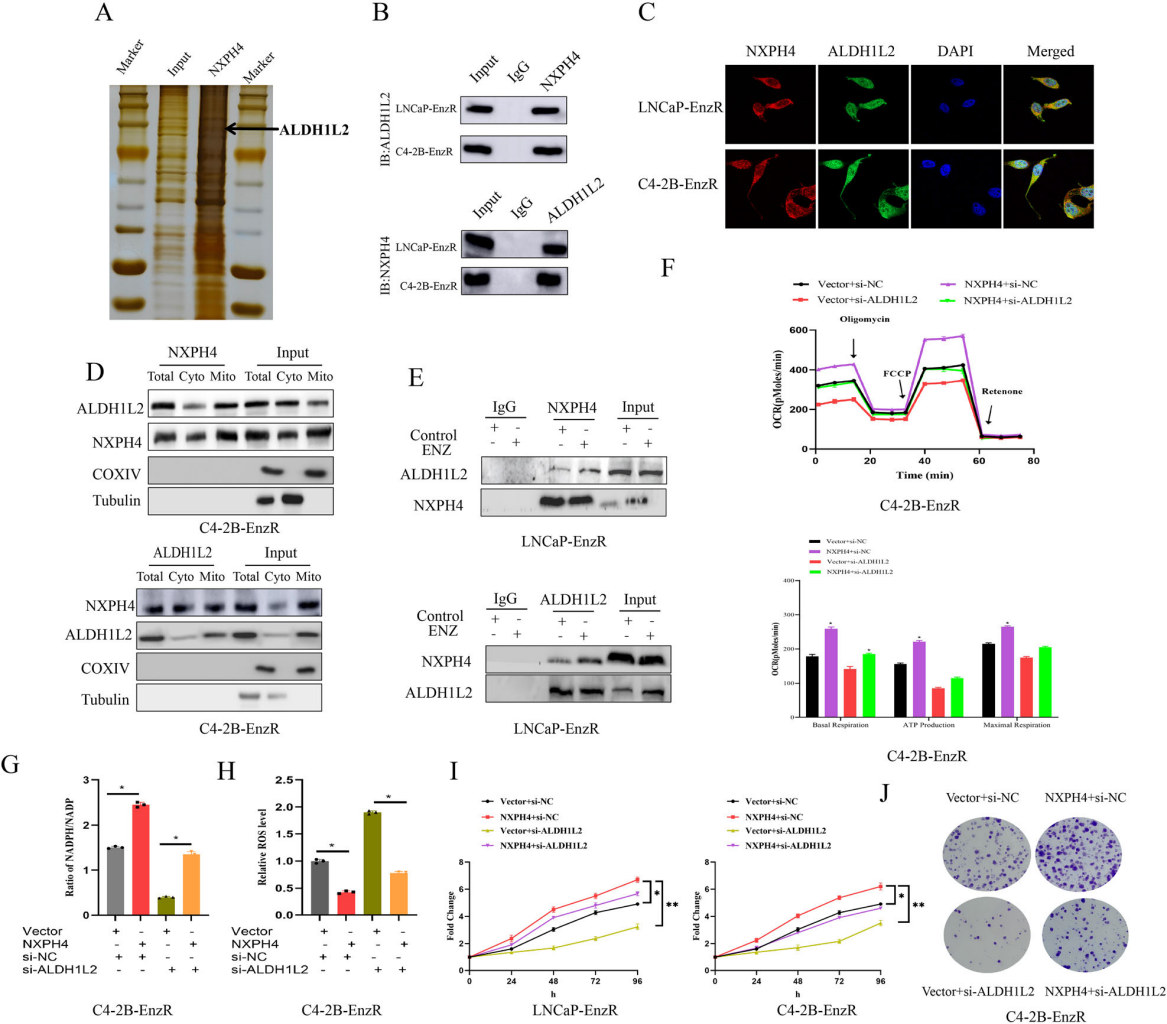
NXPH4 enhances the mitochondrial function via ALDH1L2. **A**, **B** Identifcation of proteins that interact with NXPH4 by co-IP assays and mass spectrometry analysis. C4-2B cell lysates were immunoprecipitated with NXPH4 antibody and subjected to SDS-PAGE. **C** The distribution of NXPH4 (red) and ALDH1L2 (green) was analyzed by immunofluorescence staining in C4-2B-EnzR and LNCaP-EnzR cells. **D** The binding potential between NXPH4 and ALDH1L2 in the whole cell lysates (Total), cytoplasmic fraction (Cyto) and mitochondrial fraction (Mito) were performed by co-IP assays in C4-2B cells. IgG serves as negative control. COXIV and tubulin were used as mitochondrial and cytosolic markers. **E** The binding potential between NXPH4 and ALDH1L2 was analyzed by co-IP assays in LNCaP cells with or without enzalutamide. IgG serves as negative control. **F** Measurement of OCR in C4-2B cells transfected with NXPH4 overexpression plasmid for 24 h and subsequent ALDH1L2 siRNA for another 24 h. Representative recordings of OCR during extracellular flow Seahorse analysis are shown in the up panel, and quantitative analysis of the calculated basal and maximum respiratory rates, ATP production rate and spare respiratory capacity are shown in the bottom panel. Measurement of NADPH/NADP^+^ ratio (**G**) and ROS levels (**H**) in C4-2B cells transfected with NXPH4 overexpression plasmid for 24 h and subsequent ALDH1L2 siRNA for another 24 h. **I**, **J** Cell viability and cell clone formation assays were performed in C4-2B cells co-transfected with NXPH4 overexpression plasmid for 24 h and subsequent ALDH1L2 siRNA for another 24 h. ENZ Enzalutamide. EnzR Enzalutamide Resistance. *P < 0.05, **P < 0.01.

For investigation the mechanism by which NXPH4 regulates mitochondrial function, we performed Co-IP with NXPH4 antibodies. Proteins that co-precipitated with NXPH4 were then identified using mass spectrometry. Based on their peptide count and relevance to cellular metabolism in the list of candidate proteins, we first analyzed the interactome of NXPH4 and identified ALDH1L2 as a potential mitochondrial interactor (Supplementary Table S4). Co-IP assays and immunofluorescence staining confirmed that NXPH4 physically interacts with ALDH1L2 in EnzR cells (Fig. 5A–C). Proximity ligation assay (PLA) also showed that the interaction between NXPH4 and ALDH1L2 (Supplementary Fig. 4A). To further elucidate the subcellular localization of this interaction, cytoplasmic and mitochondrial extracts were isolated from C4-2B cells and subjected to co-IP assays. Notably, the NXPH4-ALDH1L2 complex was predominantly localized in the mitochondria (Fig. 5D). Additionally, we revealed that the interaction between NXPH4 and ALDH1L2 was enhanced in response to enzalutamide treatment (Fig. 5E). These findings suggest that the NXPH4-ALDH1L2 complex plays a significant role in mitochondrial function and is modulated by enzalutamide.

We further demonstrated that siRNA-mediated knockdown of ALDH1L2 attenuated the increased OCR and NADPH/NADP^+^ ratio, while restoring the reduced ROS production induced by NXPH4 overexpression (Fig. 5F–H, Supplementary Fig. 4B). Additionally, the enhanced cell proliferation and increased colony formation resulting from NXPH4 overexpression were significantly diminished upon ALDH1L2 silencing (Fig. 5I, J). To further elucidate the molecular mechanisms by which NXPH4 regulates ALDH1L2 and its impact on mitochondrial function, we investigate whether NXPH4 influences the stability and enzymatic activity of ALDH1L2. The cycloheximide (CHX) assays was performed to examine ALDH1L2 protein stability after NXPH4 knockdown. The results indicate that NXPH4 prolongs the half-life of ALDH1L2, supporting a role in post-translational stabilization. Additionally, the ALDH1L2 enzymatic activity was evaluated, which decreased significantly after NXPH4 knockdown. These data indicate that NXPH4 supports ALDH1L2 stability and activity to maintain mitochondrial NADPH production and redox homeostasis (Supplementary Fig. 4C and D). Taken together, these findings indicate that NXPH4 enhances mitochondrial function in PCa cells through its interaction with ALDH1L2, highlighting the critical role of the NXPH4-ALDH1L2 axis in regulating mitochondrial activity.

### Targeting NXPH4 suppresses EnzR tumor growth in vivo

**Fig. 6:**
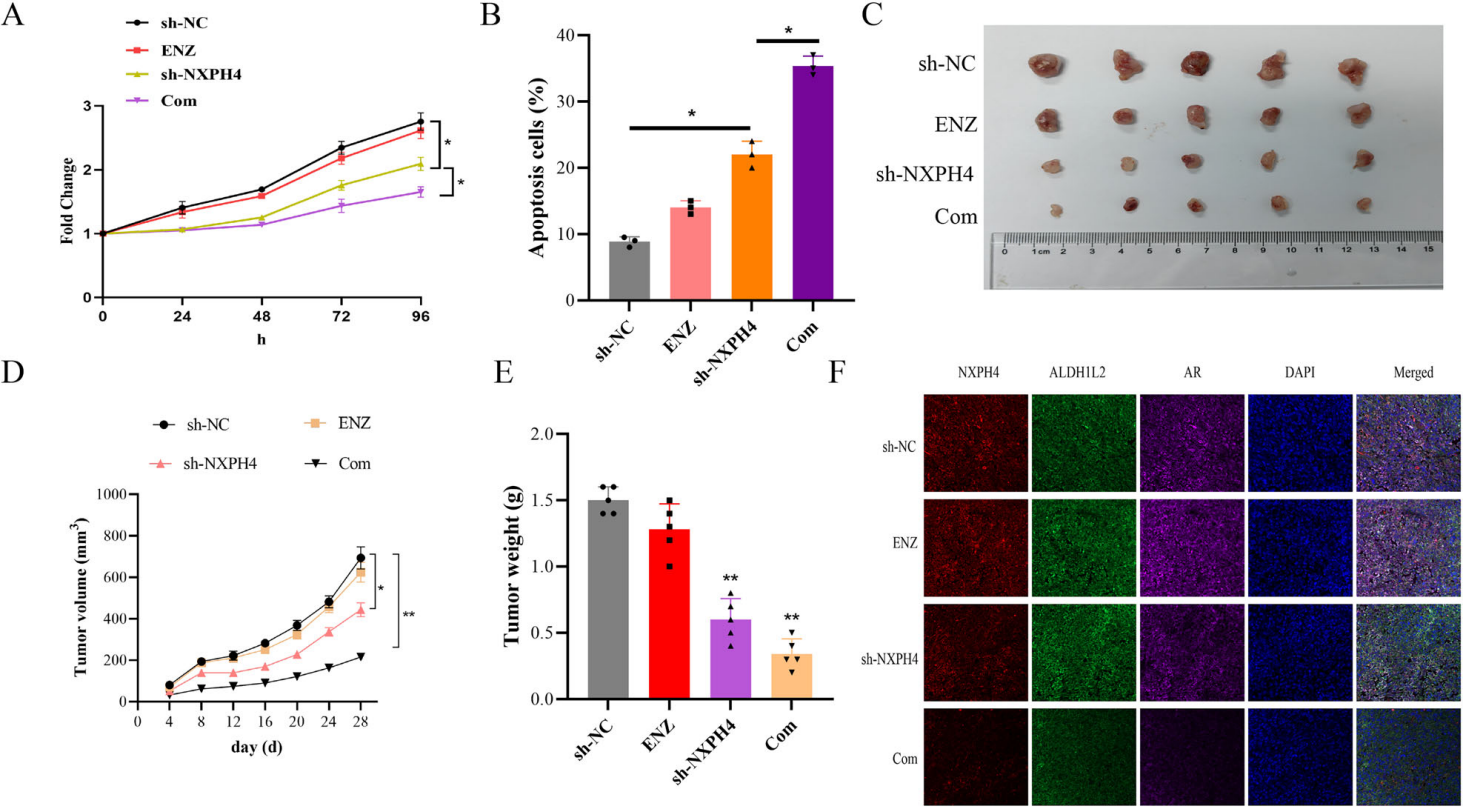
Targeting NXPH4 suppresses EnzR tumor growth in vivo. **A** Cell proliferation assessed by CCK-8 assay and cell apoptosis assay (**B**) in indicated cells. **C**–**E** Images of excised xenograft tumors from differenttreatment groups. Immunofluorescence staining for NXPH4, ALDH1L2 and AR in xenograft tumors as indicated (**F**). ENZ Enzalutamide. Com Combination. *P < 0.05, **P < 0.01.

The above results indicate that NXPH4 plays a significant role in CRPC. We speculate that blocking the NXPH4 axis, followed by combination with enzalutamide, might effectively inhibit the progression of CRPC. As shown in Fig. 6A, B, in C4-2B-EnzR cells, knocking down NXPH4 in conjunction with enzalutamide significantly inhibits cell proliferation and markedly induces apoptosis. To validate the role of NXPH4 in enzalutamide-resistant PCa in vivo, we established a xenograft mouse model using C4-2B-EnzR cells. The enzalutamide-treated group and the control group exhibited no significant differences in tumor volume or weight, confirming the resistance of these cells to enzalutamide. In contrast, the group with NXPH4 knockdown demonstrated a significant reduction in both tumor volume and weight compared to the control group. Furthermore, the combination of NXPH4 knockdown and enzalutamide treatment resulted in even greater reductions in tumor volume and weight (Fig. 6C–F). The orthotopic model also demonstrated that knockdown of NXPH4 inhibits tumor growth (supplementary Fig. 5). These results suggest that NXPH4 deletion not only inhibits the proliferation of C4-2B-EnzR cells in vitro but also in vivo, underscoring the potential of NXPH4 as a promising biomarker and therapeutic target for enzalutamide-resistant PCa.

## Discussion

Our present study elucidates a potential mechanism underlying enzalutamide resistance, involving NXPH4 overexpression and the activation of mitochondrial metabolism in PCa. We found that NXPH4 is significantly overexpressed in enzalutamide-resistant cell models. Inhibition of NXPH4 reduced cell viability and enhanced sensitivity to enzalutamide. These findings demonstrate that NXPH4 overexpression and the activation of mitochondrial metabolism play a critical role in enzalutamide resistance. Targeting the NXPH4/ALDH1L2 axis may represent a promising strategy to improve the efficacy of enzalutamide in PCa treatment.

The multifaceted functionality of mitochondria endows tumor cells with remarkable adaptive capabilities, enabling them to survive in hostile environments [18, 19]. Consequently, mitochondrial escape mechanisms must be thoroughly considered in cancer treatment strategies. Research on combined mitochondrial intervention therapies holds significant importance for advancing cancer therapeutics [20, 21]. Mitochondria have emerged as a prominent therapeutic target in oncology, with an increasing number of mitochondrial-targeted anticancer drugs currently under development [22, 23].

In PCa, a close interplay exists among the AR signaling pathway, mitochondrial morphology, and metabolic reprogramming [24, 25]. Studies have demonstrated that a mitochondria-rich phenotype is strongly associated with disease progression and poor patient prognosis [26]. Furthermore, androgen deprivation in PCa cells disrupts mitochondrial membrane integrity, and impairments in mitochondrial function or structure can trigger Ca²⁺ overload and oxidative stress, ultimately leading to cell death in CRPC [27]. Based on these findings, we hypothesize that enhanced mitochondrial activity, particularly under androgen-deprived conditions, may promote resistance to ADT and drive CRPC progression [28]. Consequently, mitochondrial-targeted therapeutic strategies could offer a promising avenue to overcome treatment resistance and suppress CRPC advancement.

NXPH4 is an important member of the neurexophilin family. Research has shown that NXPH4 plays a tumor-promoting role in various cancers. In lung cancer, NXPH4 enhances tumor cell proliferation and migration [15]. Additionally, studies on liver cancer and colorectal cancer have demonstrated that NXPH4 is closely associated with poor patient prognosis and may contribute to tumor progression [13, 29]. In bladder cancer, NXPH4 has been found to induce gemcitabine resistance, a process closely linked to the reprogramming of glycolytic metabolism [14, 30]. These findings suggest that NXPH4 may play a significant role in the initiation, progression, and treatment resistance of multiple cancers, warranting further in-depth investigation into its underlying mechanisms. Analysis of public databases revealed a positive correlation between NXPH4 and PSA expression at the mRNA level. To investigate whether NXPH4 expression is transcriptionally regulated by the AR, we performed ChIP and dual-luciferase reporter assays, confirming that AR binds to the NXPH4 promoter region and exerts an enhancing transcriptional regulatory effect on NXPH4 expression. Functional studies demonstrated that NXPH4 knockdown significantly suppressed prostate cancer cell proliferation, promoted apoptosis, and enhanced enzalutamide sensitivity. Conversely, NXPH4 overexpression alleviated androgen deprivation-induced growth inhibition and apoptosis in PCa cells and reduced enzalutamide sensitivity, findings that were further validated in in vivo experiments. Our current subcutaneous model effectively captures the EnzR phenotype. To build on this foundation, future work will employ orthotopic models to further confirm the role of NXPH4 under clinically relevant conditions.

To elucidate the mechanism of NXPH4, RNA-seq analysis revealed that NXPH4-regulated differentially expressed genes were primarily enriched in metabolism-related pathways. Additionally, co-IP coupled with mass spectrometry identified NXPH4-interacting proteins that were predominantly localized to mitochondria. Functional investigations showed that NXPH4 overexpression significantly reversed the decline in mitochondrial OCR induced by enzalutamide treatment, whereas NXPH4 silencing exacerbated enzalutamide-mediated OCR reduction. Furthermore, assessments of mitochondrial membrane potential, ROS levels, and the NADPH/NADP^+^ ratio demonstrated that NXPH4 overexpression enhanced mitochondrial functional activity under androgen-deprived conditions, while NXPH4 knockdown led to a marked decrease in mitochondrial activity. These results indicate that NXPH4 promotes metabolic adaptation and survival of PCa cells by regulating mitochondrial function, thereby contributing to enzalutamide resistance. NXPH4 may serve as a potential therapeutic target for PCa treatment.

We conducted a systematic screening of NXPH4-interacting proteins and successfully identified ALDH1L2 as a key potential interacting partner of NXPH4. ALDH1L2 is a critical enzyme in mitochondrial one-carbon metabolism, playing important roles in NADPH production and antioxidant defense [31, 32]. Experimental validation confirmed the specific binding between NXPH4 and ALDH1L2 in cells, with their interaction primarily occurring in mitochondrial compartments. Functional studies demonstrated that ALDH1L2 knockdown significantly attenuated the mitochondrial function enhancement induced by NXPH4 overexpression, suggesting that NXPH4 may regulate mitochondrial function by modulating ALDH1L2 activity. The NXPH4-ALDH1L2 protein interaction represents a critical regulatory axis in mitochondrial function, and therapeutic targeting of this signaling pathway effectively overcomes enzalutamide resistance in prostate cancer. Although this study did not identify a specific inhibitor of NXPH4, future work will focus on structural modeling and small-molecule screening to identify compounds that interfere with NXPH4-ALDH1L2 binding, which may offer a new therapeutic approach to overcome enzalutamide resistance in PCa. Enzalutamide has been reported to perturb mitochondrial function and increase oxidative stress in PCa cells. Mitochondrial perturbation can trigger the redistribution of cytosolic factors to mitochondria as part of adaptive stress responses; thus, NXPH4 translocation may be a downstream consequence of enzalutamide-induced mitochondrial stress.

In conclusion, our findings demonstrate that NXPH4 drives enzalutamide resistance by reprogramming mitochondrial metabolism through ALDH1L2-dependent mechanisms. Therapeutically, combined targeting of NXPH4 and enzalutamide treatment synergistically suppresses CRPC growth, suggesting a promising dual-targeting strategy for advanced prostate cancer.

## Methods

### Cell culture

LNCaP and C4-2B cell lines were obtained from the American Type Culture Collection (ATCC; Manassas, VA, USA) and maintained according to ATCC protocols. Enzalutamide-resistant cell lines were established through continuous culture in medium containing 10 µM enzalutamide for a minimum of 6 months. For androgen deprivation studies, PCa cells were cultured in phenol red-free medium supplemented with 10% charcoal-stripped fetal bovine serum to achieve hormone-starved conditions.

### Quantitative Real-Time Polymerase Chain Reaction (qRT-PCR)

Total RNA was extracted from cells and tissues following the manufacturer’s instructions. RNA concentration and purity were measured using an ultraviolet spectrophotometer at 260/280 nm. cDNA was synthesized from RNA using a PrimerScript One-Step RT-PCR Kit (TaKaRa). Real-time PCR analyses were performed using SYBR Green on a Real-Time PCR System (Applied Biosystems, CA, USA). Gene expression levels were normalized to GAPDH and calculated using the 2^−△△Ct^ method. Primer sequences were synthesized by Sangon Biotech (Shanghai, China) and are listed in Table S1. All experiments were conducted in triplicate.

### Western blot assay

Briefly, protein samples extracted from cells or tissues were denatured, separated by electrophoresis on 10% polyacrylamide gels, and transferred to polyvinylidene difluoride (PVDF) membranes (Millipore, Burlington, MA, USA). The membranes were then incubated with primary antibodies. Protein band intensities were quantified using ImageJ software (NIH, Maryland, USA). The information of antibodies is summarized in Supplementary Table 2.

### Cell transfection

Lentiviruses containing control shRNA (NC) and specific shRNA against NXPH4 were infected into PCa cells according to the manufacturer’s instructions. Cells were infected for 24 h with medium containing virus and 1 ng/mL polybrene (Sigma, USA). The transduced cells were then screened for 3 days with puromycin (Sigma) at a concentration of 2 mg/mL. The plasmid was transfected into cells with lipofectamine 3000 (Invitrogen, USA).

### RNA sequencing (RNA‑seq) and bioinformatics analysis

Total RNA was isolated from control (sh-NC) and NXPH4 knockdown (sh-NXPH4) cells and RNA integrity was confirmed on an Agilent Bioanalyzer. Libraries were prepared with the Illumina TruSeq Stranded mRNA Library Prep Kit and sequenced as 2 × 150 bp reads on an Illumina NovaSeq 6000 to a target depth of 30–50 M read pairs/sample. Raw reads were quality-checked using FastQC. Differential expression analysis was performed with DESeq2 and genes with adjusted *p* < 0.05 and |log2FC | ≥ 1 were considered significant.

### Proximity ligation assay (PLA)

The interaction between NXPH4 and ALDH1L2 was further validated using a Duolink® in situ PLA kit (Sigma-Aldrich) according to the manufacturer’s instructions. Briefly, cells were seeded onto glass coverslips and fixed with 4% paraformaldehyde for 15 min at room temperature, followed by permeabilization with 0.2% Triton X-100 for 10 min. After blocking, the cells were incubated overnight at 4 °C with primary antibodies against NXPH4 and ALDH1L2 derived from different species. Subsequently, cells were incubated with species-specific PLA probes (PLUS and MINUS) for 1 h at 37 °C. Ligation and amplification reactions were performed using the Duolink® reagents, and the resulting PLA signals (red fluorescent puncta) were visualized under a fluorescence microscope. DAPI was used to stain nuclei.

### Flow cytometry analysis

Flow cytometry was performed to assess the effect of NXPH4 knockdown on cell death and cell cycle progression. Briefly, cells were harvested and washed twice with cold PBS, then resuspended in PBS containing 2% FBS to prevent nonspecific binding. For apoptosis detection, cells were incubated with Annexin V-FITC and propidium iodide (PI) following the manufacturer’s instructions for 15 min at room temperature in the dark. The samples were analyzed on a BD flow cytometer.

### Chromatin Immunoprecipitation (ChIP) assay

ChIP assays were performed to examine the binding of AR to the NXPH4 promoter. Briefly, cells were crosslinked with 1% formaldehyde for 10 minutes at room temperature, and the reaction was quenched with 125 mM glycine for 5 min. Cells were then lysed and the chromatin was sheared to fragments of approximately 200–500 bp using sonication. The lysates were incubated overnight at 4 °C with anti-AR antibody or normal IgG (as a negative control), followed by precipitation with protein A/G magnetic beads. After sequential washing, the crosslinks were reversed by heating at 65 °C for 6 h, and DNA was purified using a phenol–chloroform extraction or commercial purification kit. The enrichment of specific DNA fragments was analyzed by qPCR using primers targeting the NXPH4 promoter region containing the putative AR-binding site.

### JC-1 staining assay

Briefly, cells were seeded in 6-well plates and treated according to the indicated conditions. After treatment, cells were incubated with JC-1 dye (5 μM, Beyotime, China) at 37 °C for 20 min in the dark. The cells were then washed twice with JC-1 buffer to remove excess dye and immediately analyzed using fluorescence microscopy. The red/green fluorescence intensity ratio was calculated to quantify changes in mitochondrial membrane potential.

### Tissue sample

Prostate cancer tissue samples were collected from patients undergoing urological surgery at the Second Affiliated Hospital of Anhui Medical University. All procedures in this study were approved by the Ethics Committee of the Second Affiliated Hospital of Anhui Medical University, and written informed consent was obtained from each participant prior to sample collection.

### Xenograft studies in nude mice

Male nude mice aged 4–6 weeks were obtained from Weitonglihua Biotechnology (Beijing, China). Briefly, 1 × 10⁷ C4-2B cells expressing negative control sh-RNA (sh-NC) or sh-NXPH4 were resuspended in 100 μL of phosphate-buffered saline (PBS) mixed with 50% Matrigel and injected subcutaneously into the mice. Tumor volume was calculated using the formula (length × width² × 0.5) and measured twice weekly. When tumor volumes reached approximately 300 mm³, the tumor-bearing mice underwent castration. Tumor size was measured every four days, and on day 28, the mice were euthanized to measure tumor weight. All animal experiments were conducted in accordance with the guidelines of the Ethics Committee of Anhui Medical University.

### Mitochondrial respiration

Mitochondrial respiration was assessed using an XF96 extracellular flux analyzer (Seahorse Biosciences, Agilent Technologies, Santa Clara, CA, USA) following protocols. Briefly, cells were seeded in an XFe96 cell culture microplate and allowed to adhere. The oxygen consumption rate (OCR) was measured under basal conditions and following sequential injection of mitochondrial stress test compounds: oligomycin (1 μM) to assess ATP-linked respiration, carbonyl cyanide-4-(trifluoromethoxy)phenylhydrazone (FCCP, 1 μM) to measure maximal respiratory capacity, and antimycin A (0.5 μM) to determine non-mitochondrial oxygen consumption.

### Immunohistochemistry (IHC)

Antigen retrieval was performed using Tris buffer (pH 6.0) in a pressure cooker for 10 min. Tissue sections were then incubated with specified primary antibodies overnight at 4 °C. IHC staining intensity was evaluated. For quantitative assessment, staining intensity was graded semi-quantitatively using a three-tier scoring system: 0 (no staining), 1 (weak staining), 2 (moderate staining), and 3 (strong staining).

### Statistical analysis

All statistical analyses were performed using GraphPad Prism 7. Continuous variables between two groups were compared using two-tailed unpaired Student’s *t* tests. Tumor growth curves were evaluated by analysis of ANOVA. All in vitro experiments were conducted with at least three biological replicates. Data are presented as mean ± standard error of the mean. Statistical significance was defined as follows: *p < 0.05; **p < 0.01.

## Supplementary information

Supplementary legends

Table S1

Table S2

Table S3

Table S4

Original Data Western blots

Supplementary Figure 1

Supplementary Figure 2

Supplementary Figure 3

Supplementary Figure 4

Supplementary Figure 5

## Data Availability

The data that support the findings of this study are available from the corresponding author upon reasonable request.
