# Supplementary material for: Targeting NXPH4/ALDH1L2 signaling suppresses enzalutamide resistance in prostate cancer
Source: Cell Death Discov. 2026 Feb 4;12:91. doi: 10.1038/s41420-026-02944-z (PMC12894754; doi:10.1038/s41420-026-02944-z)
Supplement: Supplementary file 1 — Supplementary legends [file 41420_2026_2944_MOESM1_ESM.doc]

**Supplementary legends:**

**Supplementary Figure 1.** Effect of ENZA treatment on cell viability, colony formation, and apoptosis in enzalutamide-resistant prostate cancer cells. (A) Cell viability of LNCaP-EnzR and C4-2B-EnzR cells was assessed by CCK-8 assay after treatment with ENZ or DMSO. (B) Colony formation assay of LNCaP-EnzR and C4-2B-EnzR cells treated with ENZ or DMSO. (C) Flow cytometry analysis of apoptosis in LNCaP-EnzR and C4-2B-EnzR cells treated with ENZ or DMSO. *P<0.05, **P<0.01, NS: Not Significant.

**Supplementary Figure 2.** (A) Representative IHC staining of NXPH4 in enzalutamide-sensitive and -resistant prostate cancer tissues. NXPH4 staining intensity was markedly higher in EnzR samples. Scale bar, 50 µm. Quantification of IHC scores is shown on the right (P < 0.05). (B-C) Western blot analysis was performed to confirm the efficiency of NXPH4 knockdown and overexpression in prostate cancer cells. GAPDH was used as the internal control. (D) The relative proliferation rates for each condition (Vec+ENZ/Vec and NXPH4+ENZ/NXPH4) and compared them statistically. *P<0.05.

**Supplementary Figure 3.** (A) The mitochondrial membrane potential was evaluated using a JC-1 staining assay. JC-1 aggregates emit red fluorescence, whereas JC-1 monomers emit green fluorescence. (B) KEGG enrichment analysis showed that significant enrichment of genes associated with mitochondrial energy metabolism and oxidative stress regulation upon NXPH4 knockdown.

**Supplementary Figure 4.** (A) PLA assay of NXPH4 and ALDH1L2 in LNCaP-EnzR and C4-2B-EnzR cells. (B) Western blot analysis was performed to confirm the efficiency of ALDH1L2 knockdown. (C) Assess ALDH1L2 enzyme activity after knockdown or overexpression of NXPH4. (D) Cycloheximide (CHX) assay showing the degradation of ALDH1L2 protein in LNCaP-EnzR and C4-2B-EnzR cells with NXPH4 knockdown (sh-NXPH4). Western blots for ALDH1L2 were performed at indicated time points (0, 2, 4, 6 hours). The relative ALDH1L2 protein levels were quantified and plotted. *P<0.05, **P<0.01.

**Supplementary Figure 5.** For the orthotopic tumor model, C4-2B-EnzR cells stably expressing sh-NC or sh-NXPH4 were orthotopically injected into the anterior prostate lobes of the mice under anesthesia. Tumor growth was monitored weekly using an in vivo imaging system (IVIS Lumina ) after intraperitoneal injection of D-luciferin (150 mg/kg). After 5 weeks, mice were sacrificed, and tumors were excised, photographed, and weighed. (A) Representative bioluminescence images of C4-2B-EnzR orthotopic xenograft-bearing mice injected with sh-NC or sh-NXPH4 cells. Tumor signals were detected using an IVIS imaging system. (B) Representative images of excised tumors from each group at the endpoint of the experiment. (C) Quantification of tumor weights from the sh-NC and sh-NXPH4 groups (n = 5 per group). *P<0.05, **P<0.01.

**Supplementary Table 1.** Primer information.

**Supplementary Table 2.** Antibodies used in this study.

**Supplementary Table 3.** Differential expression metabolism related genes in RNA-seq.

**Supplementary Table 4.** Top 10 candidate proteins from mass spectrometry analysis.
