# Supplementary material for: Targeting NXPH4/ALDH1L2 signaling suppresses enzalutamide resistance in prostate cancer
Source: Cell Death Discov. 2026 Feb 4;12:91. doi: 10.1038/s41420-026-02944-z (PMC12894754; doi:10.1038/s41420-026-02944-z)
Supplement: Supplementary file 2 — Table S1 [file 41420_2026_2944_MOESM2_ESM.docx]

**Table S1: Primer information.**

| **Gene Name** | **Primer Sequence** |
| --- | --- |
| **GAPDH** | F: AATGGGCAGCCGTTAGGAAA  R: GCCCAATACGACCAAATCAGAG |
| **NXPH4** | F: TGGCTCCTTAGGAAGGCCG  R: TCCGAAGACCTTGGCTCTG |
| **ALDH1L2** | F: CTCCACTGGCCGGGTTTATT  R: AGCCAGAGGGTCAGCTTTTC |
| **AR** | F: GGTGAGCAGAGTGCCCTATC  R: GCAGTCTCCAAACGCATGTC |
| **PSA** | F: TTTCCAATGACGTGTGTGCG  R: TCAGAATGACCCACGAGCAG |
|  |  |
|  |  |
|  |  |
|  |  |

F: Forward; R: Reverse.
