# Supplementary material for: Targeting NXPH4/ALDH1L2 signaling suppresses enzalutamide resistance in prostate cancer
Source: Cell Death Discov. 2026 Feb 4;12:91. doi: 10.1038/s41420-026-02944-z (PMC12894754; doi:10.1038/s41420-026-02944-z)
Supplement: Supplementary file 6 — Original Data Western blots [file 41420_2026_2944_MOESM6_ESM.pdf]

Figure 1A

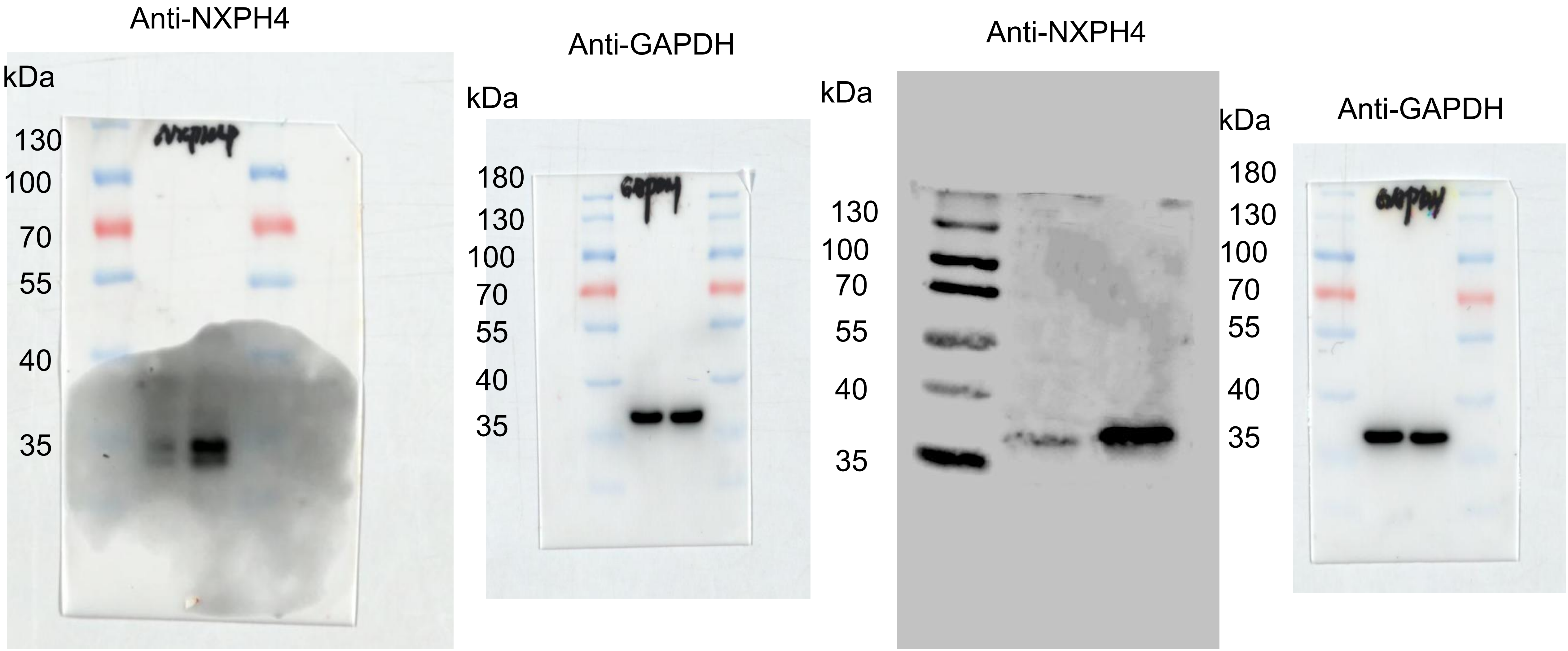

Anti-NXPH4
kDa
130
100
70
55
40
35
Anti-GAPDH
kDa
180
130
100
70
55
40
35
Anti-NXPH4
kDa
130
100
70
55
40
35
kDa
180
130
100
70
55
40
35
Anti-GAPDH

Figure 1C

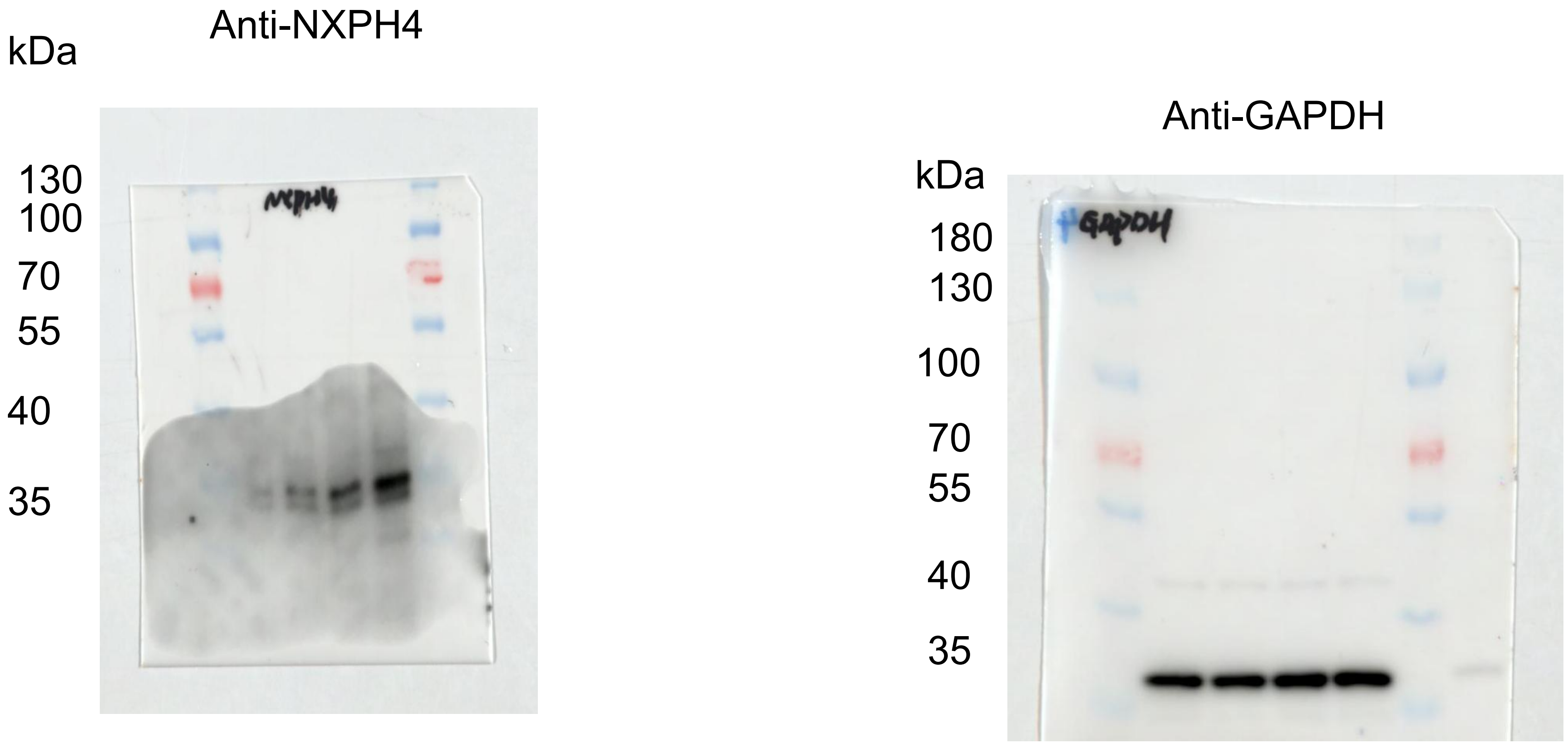
Anti-NXPH4
kDa
130
100
70
55
40
35
NXPH4
Anti-GAPDH
kDa
180
130
100
70
55
40
35
GAPDH

Figure 1C

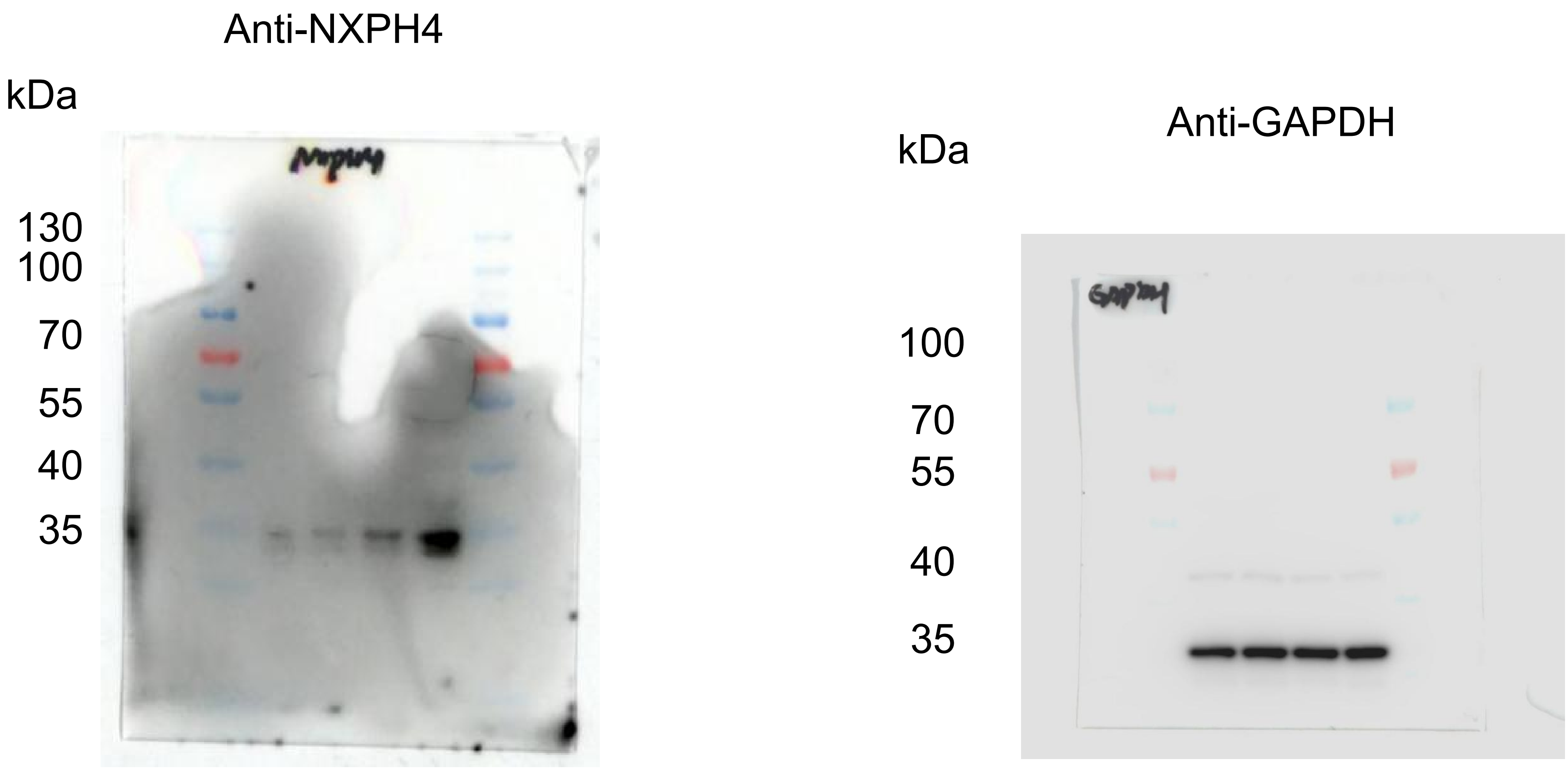

Figure 3B

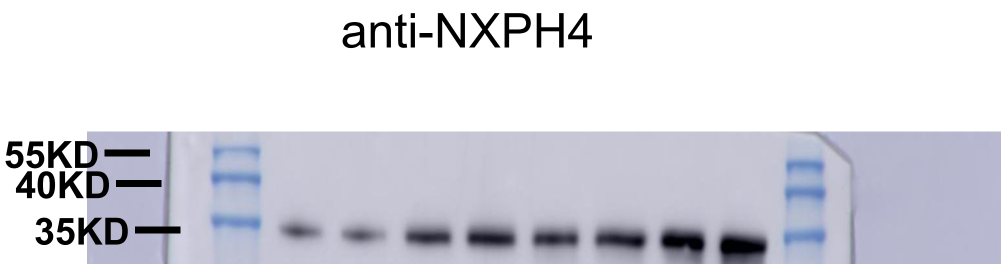

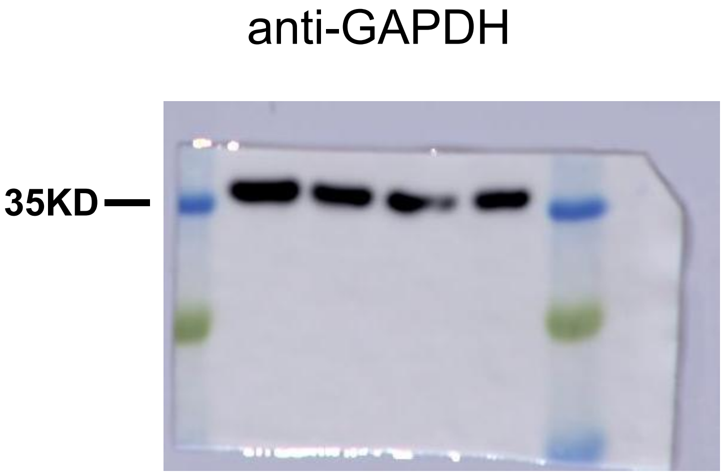

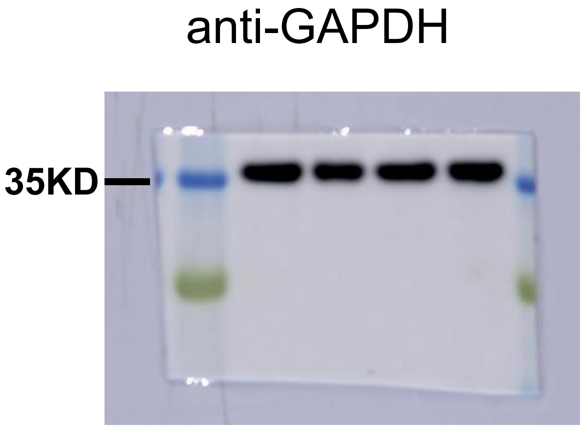

Figure 3E

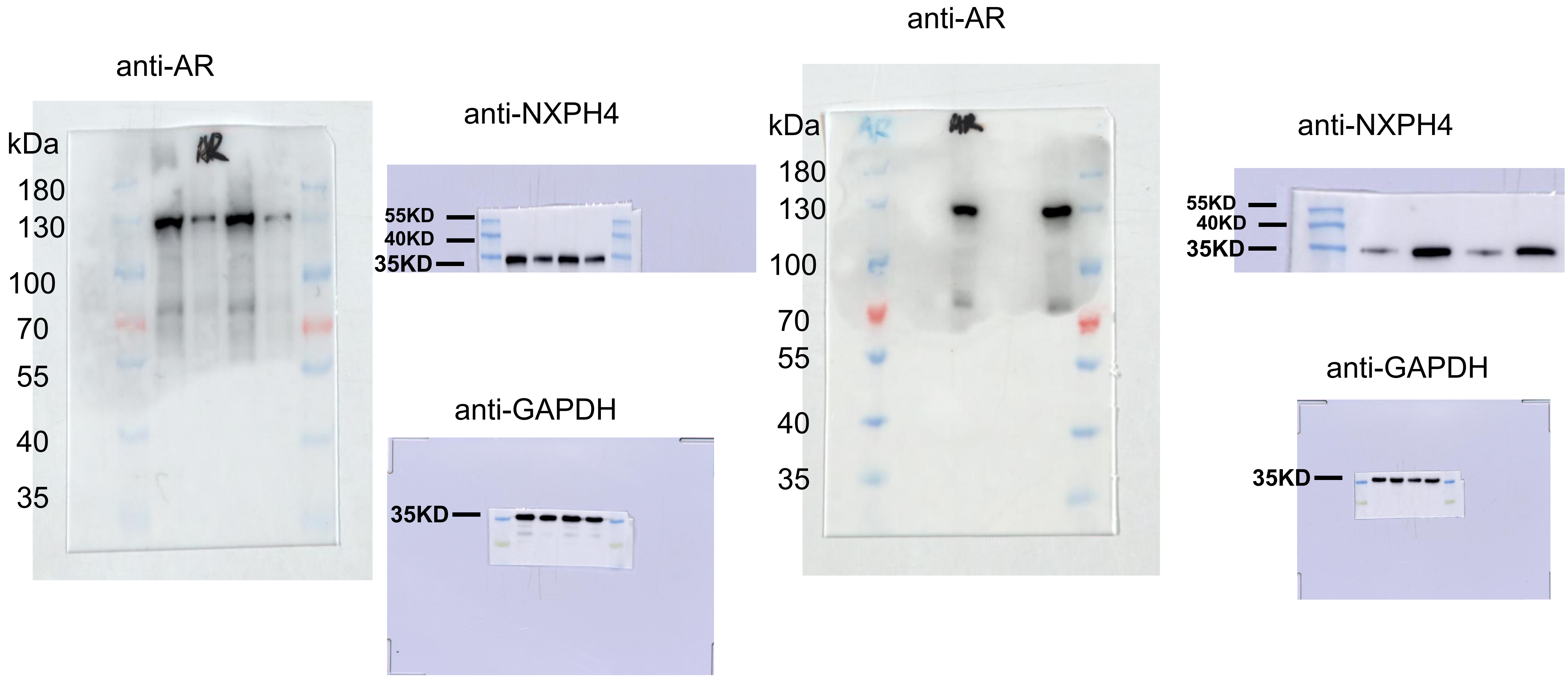
anti-AR
kDa
180
130
100
70
55
40
35
anti-NXPH4
55KD
40KD
35KD
anti-GAPDH
35KD
anti-AR
kDa
180
130
100
70
55
40
35
anti-NXPH4
55KD
40KD
35KD
anti-GAPDH
35KD

Figure 4B

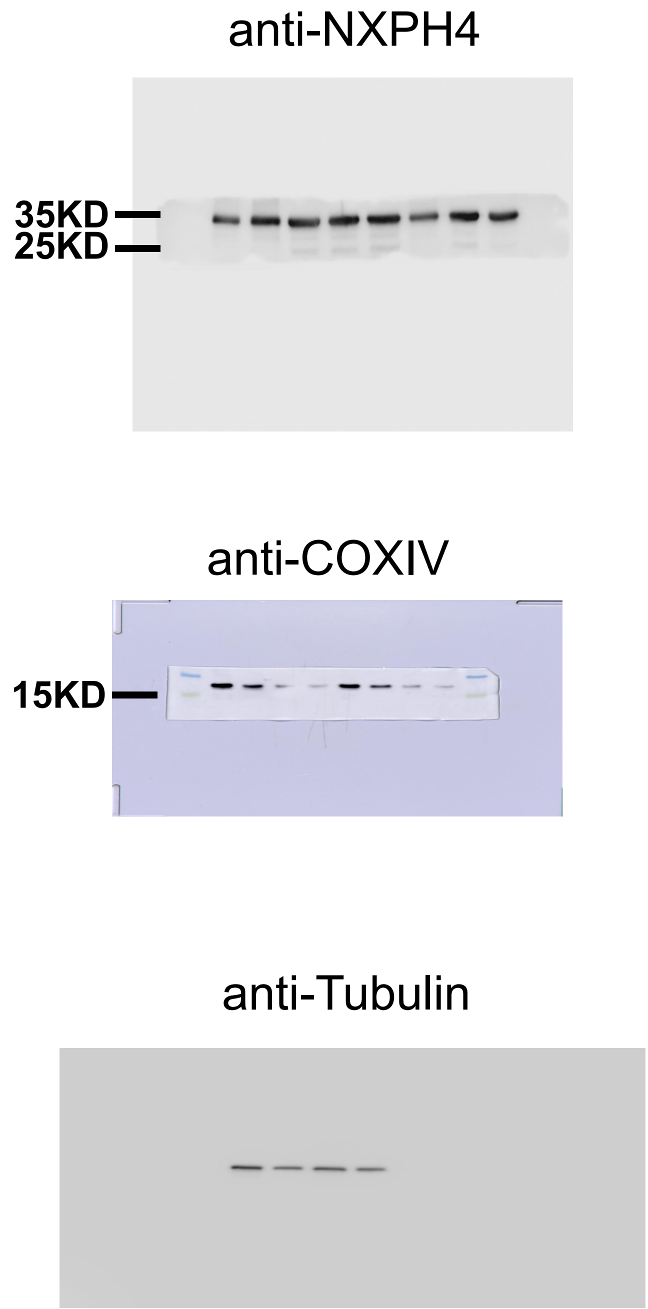

Figure 5B

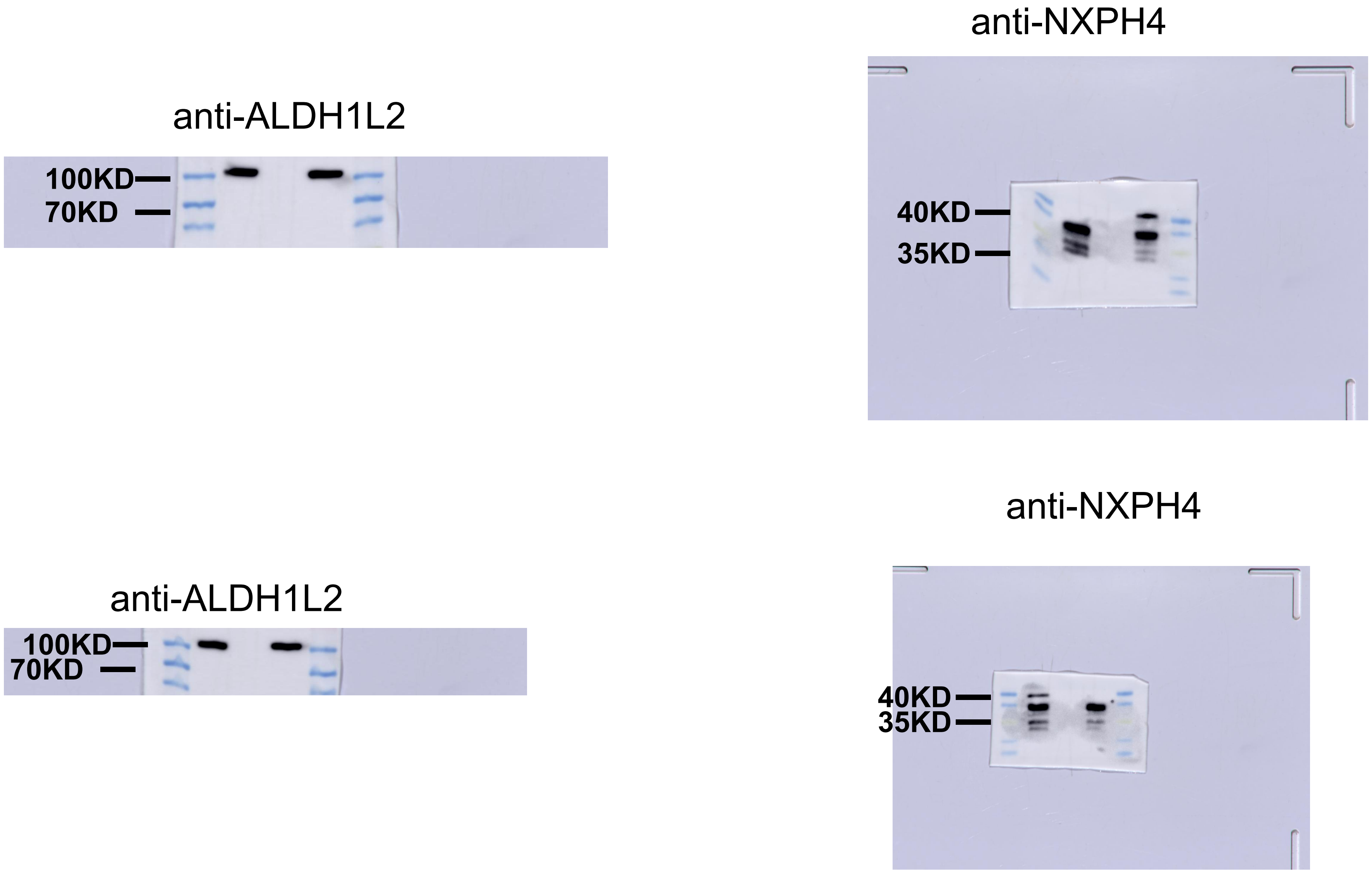
anti-NXPH4
anti-ALDH1L2
100KD
70KD
40KD
35KD
anti-NXPH4
anti-ALDH1L2
100KD
70KD
40KD
35KD

Figure 5D

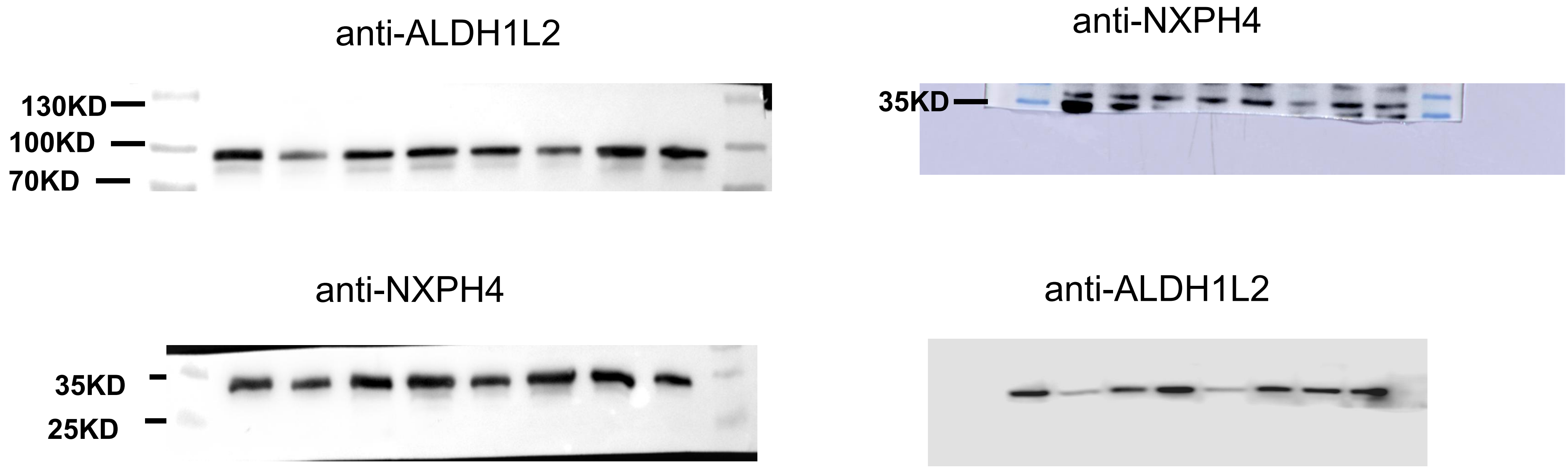
anti-ALDH1L2
130KD
100KD
70KD
anti-NXPH4
35KD
anti-NXPH4
35KD
25KD
anti-ALDH1L2

Figure 5E

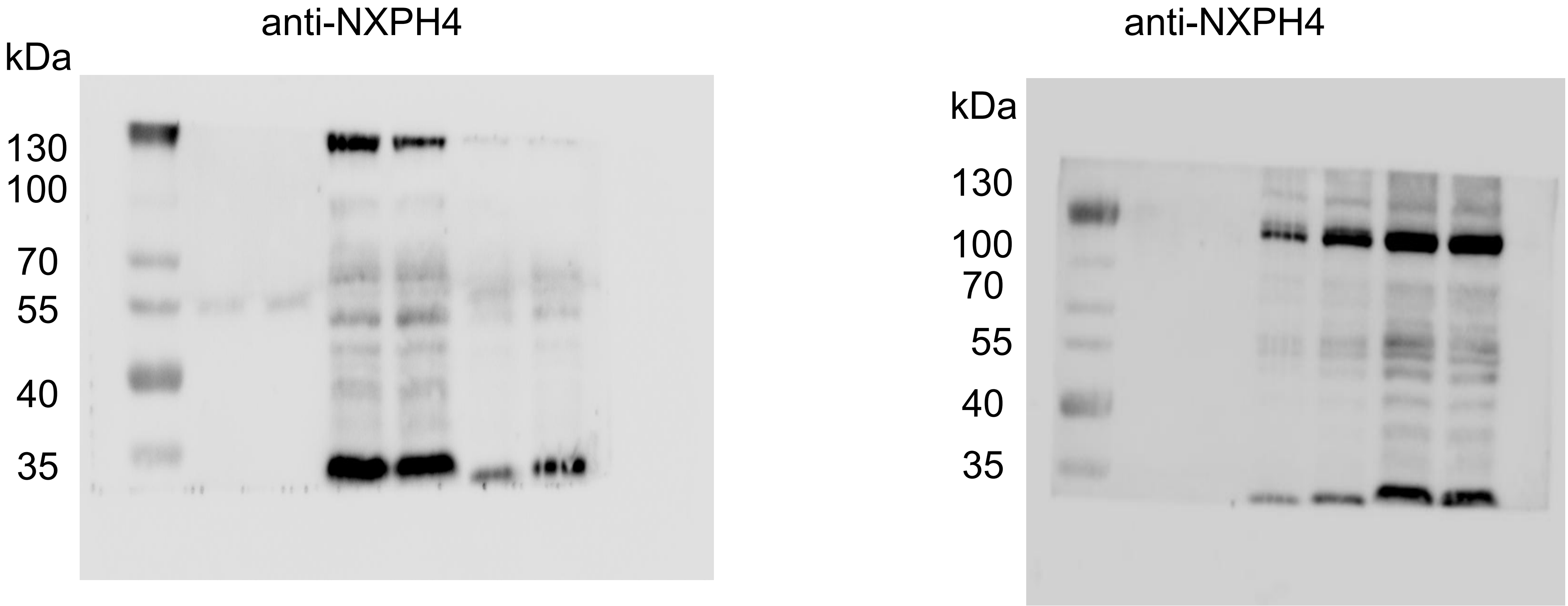
anti-NXPH4
kDa
130
100
70
55
40
35
anti-NXPH4
kDa
130
100
70
55
40
35

Figure 5E

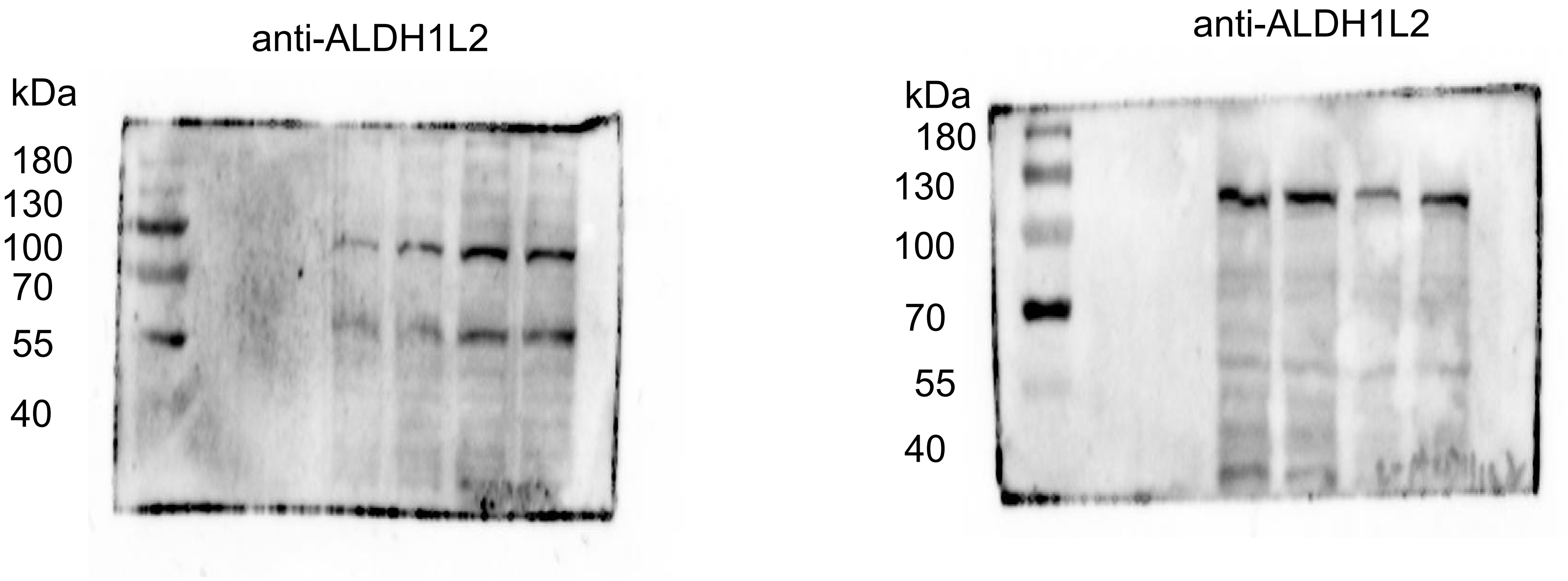
anti-ALDH1L2
kDa
180
130
100
70
55
40
anti-ALDH1L2
kDa
180
130
100
70
55
40

Figure S2C

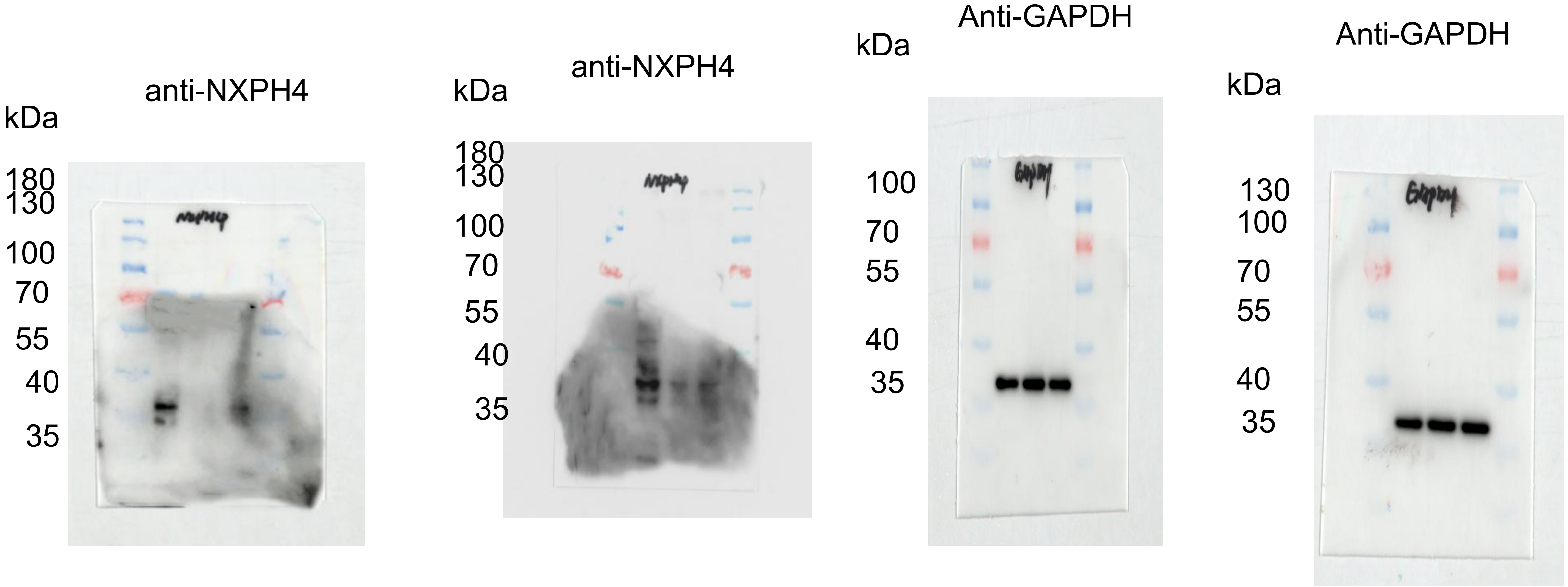
anti-NXPH4
kDa
180
130
100
70
55
40
35
anti-NXPH4
kDa
180
130
100
70
55
40
35
Anti-GAPDH
kDa
100
70
55
40
35
Anti-GAPDH
kDa
130
100
70
55
40
35

Figure S2B

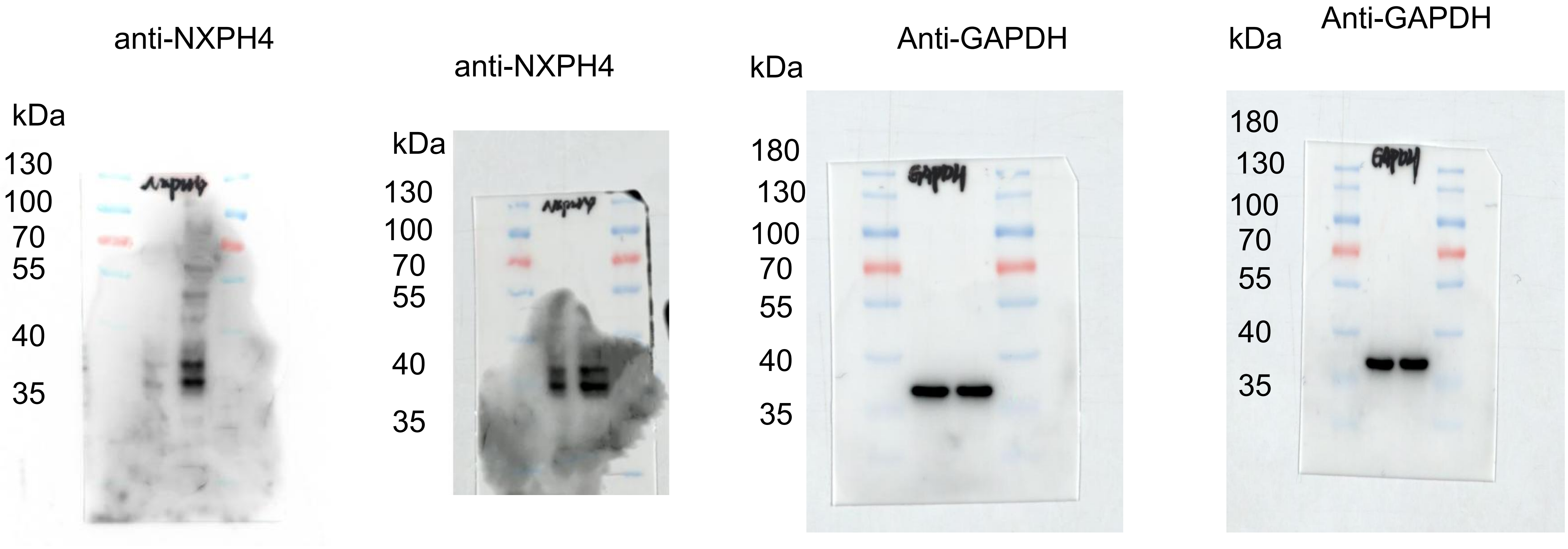
anti-NXPH4
kDa
130
100
70
55
40
35
anti-NXPH4
kDa
130
100
70
55
40
35
Anti-GAPDH
kDa
180
130
100
70
55
40
35
Anti-GAPDH
kDa
180
130
100
70
55
40
35

Figure S4B

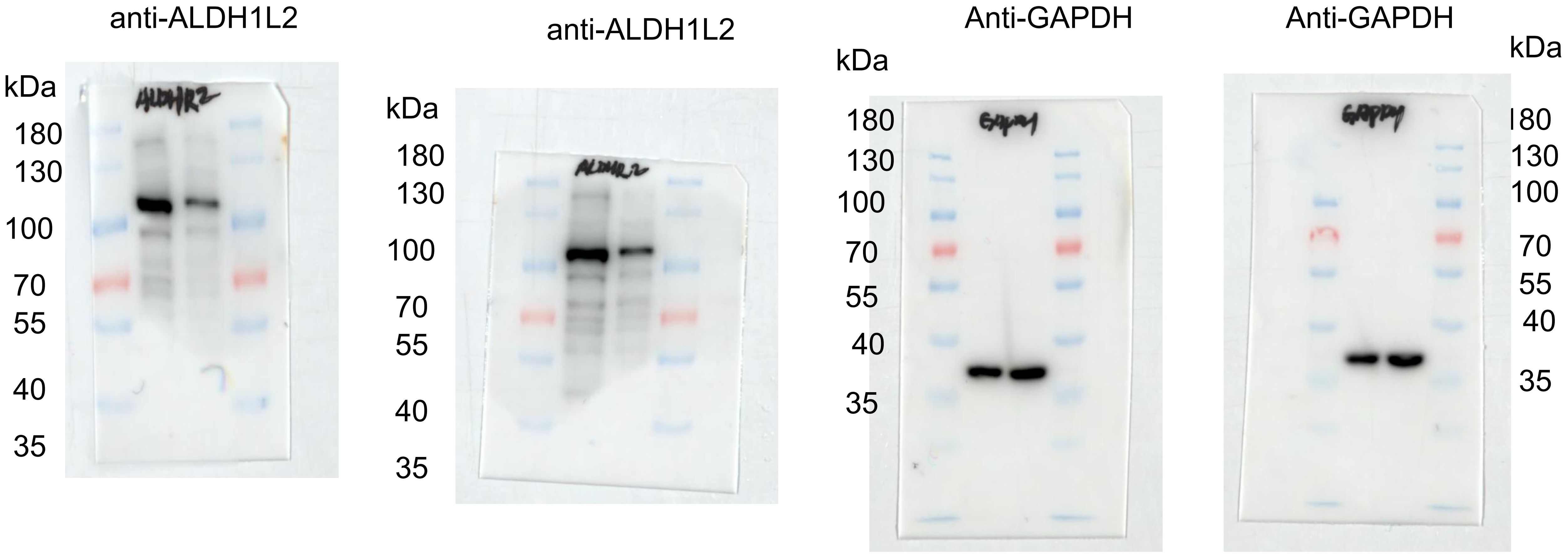
anti-ALDH1L2
kDa
180
130
100
70
55
40
35
anti-ALDH1L2
kDa
180
130
100
70
55
40
35
Anti-GAPDH
kDa
180
130
100
70
55
40
35
Anti-GAPDH
kDa
180
130
100
70
55
40
35

Figure S4D

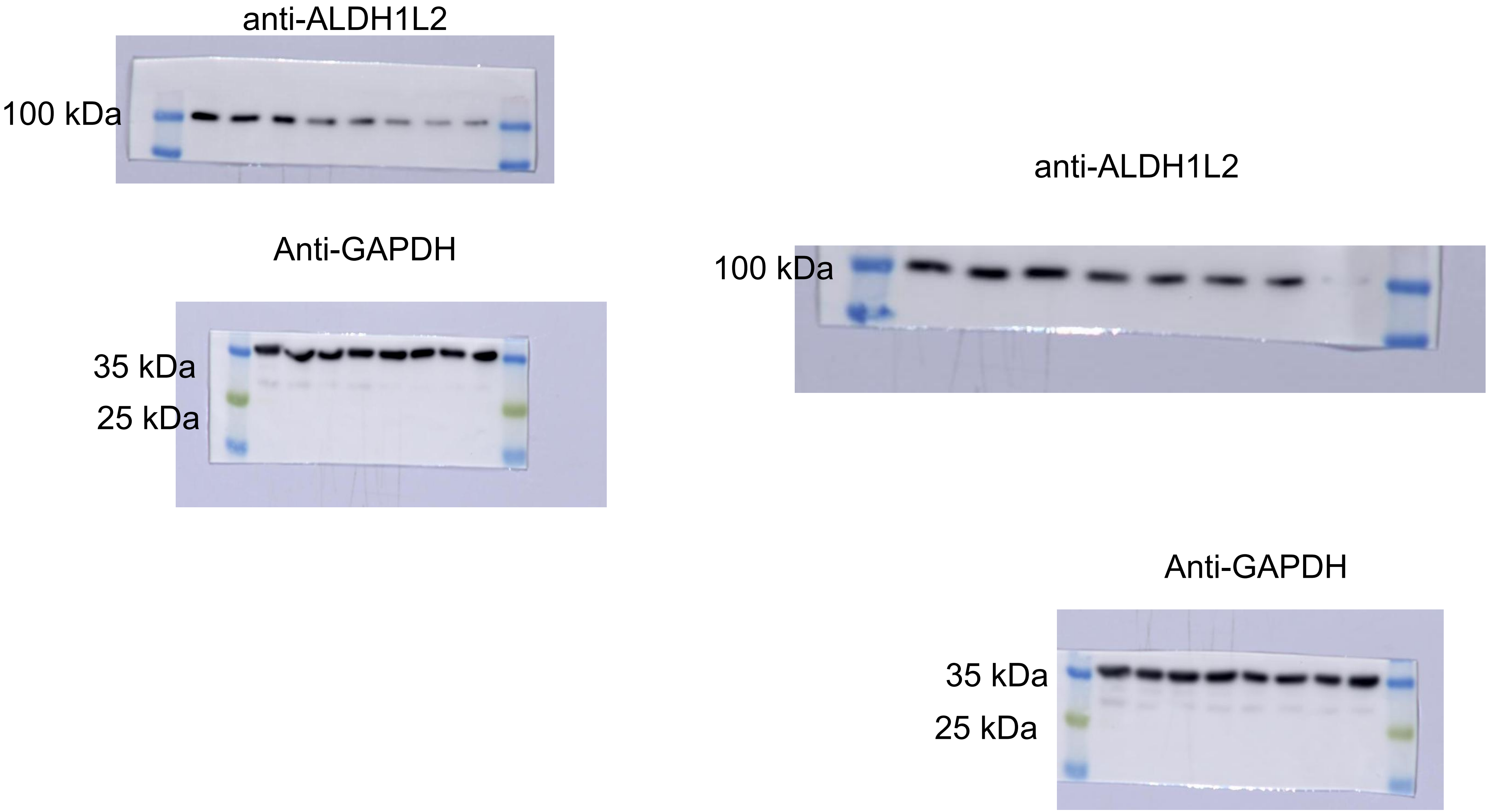
anti-ALDH1L2
100 kDa
Anti-GAPDH
35 kDa
25 kDa
anti-ALDH1L2
100 kDa
Anti-GAPDH
35 kDa
25 kDa
