# Supplementary figures and images for: Targeting NXPH4/ALDH1L2 signaling suppresses enzalutamide resistance in prostate cancer
Source: Cell Death Discov. 2026 Feb 4;12:91. doi: 10.1038/s41420-026-02944-z (PMC12894754; doi:10.1038/s41420-026-02944-z)

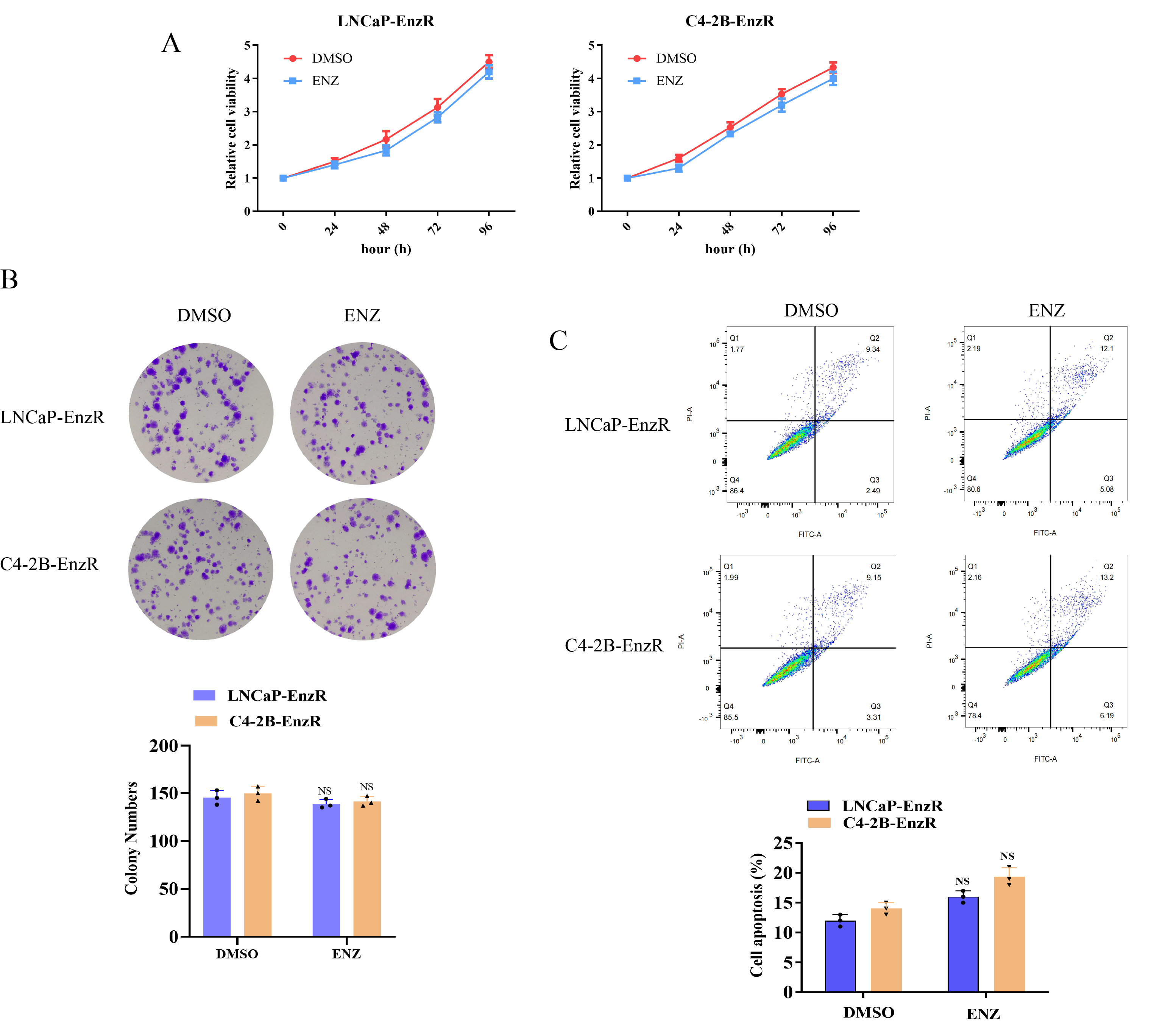

Supplement: Supplementary file 7 — Supplementary Figure 1 [file 41420_2026_2944_MOESM7_ESM.jpg]

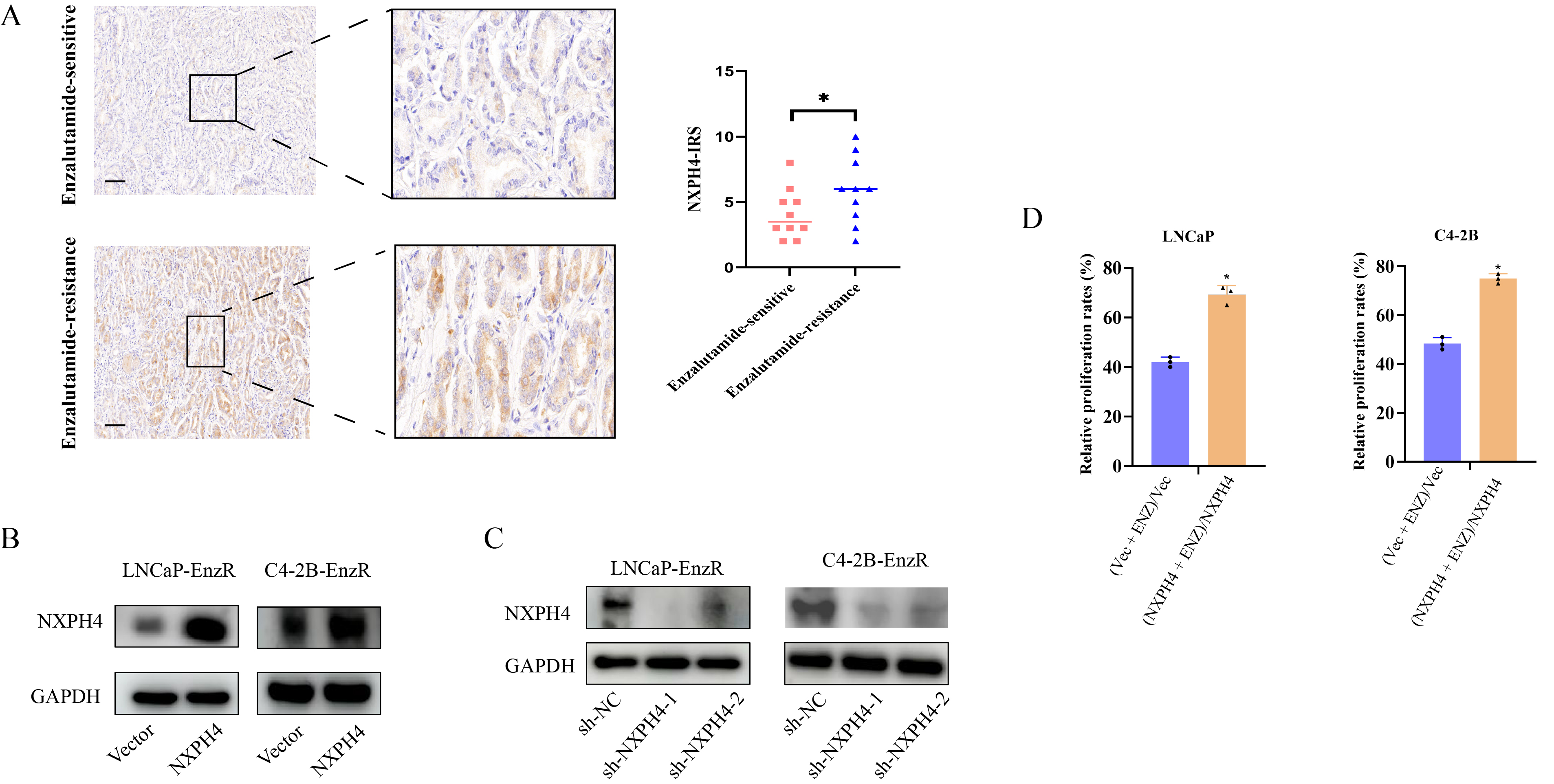

Supplement: Supplementary file 8 — Supplementary Figure 2 [file 41420_2026_2944_MOESM8_ESM.jpg]

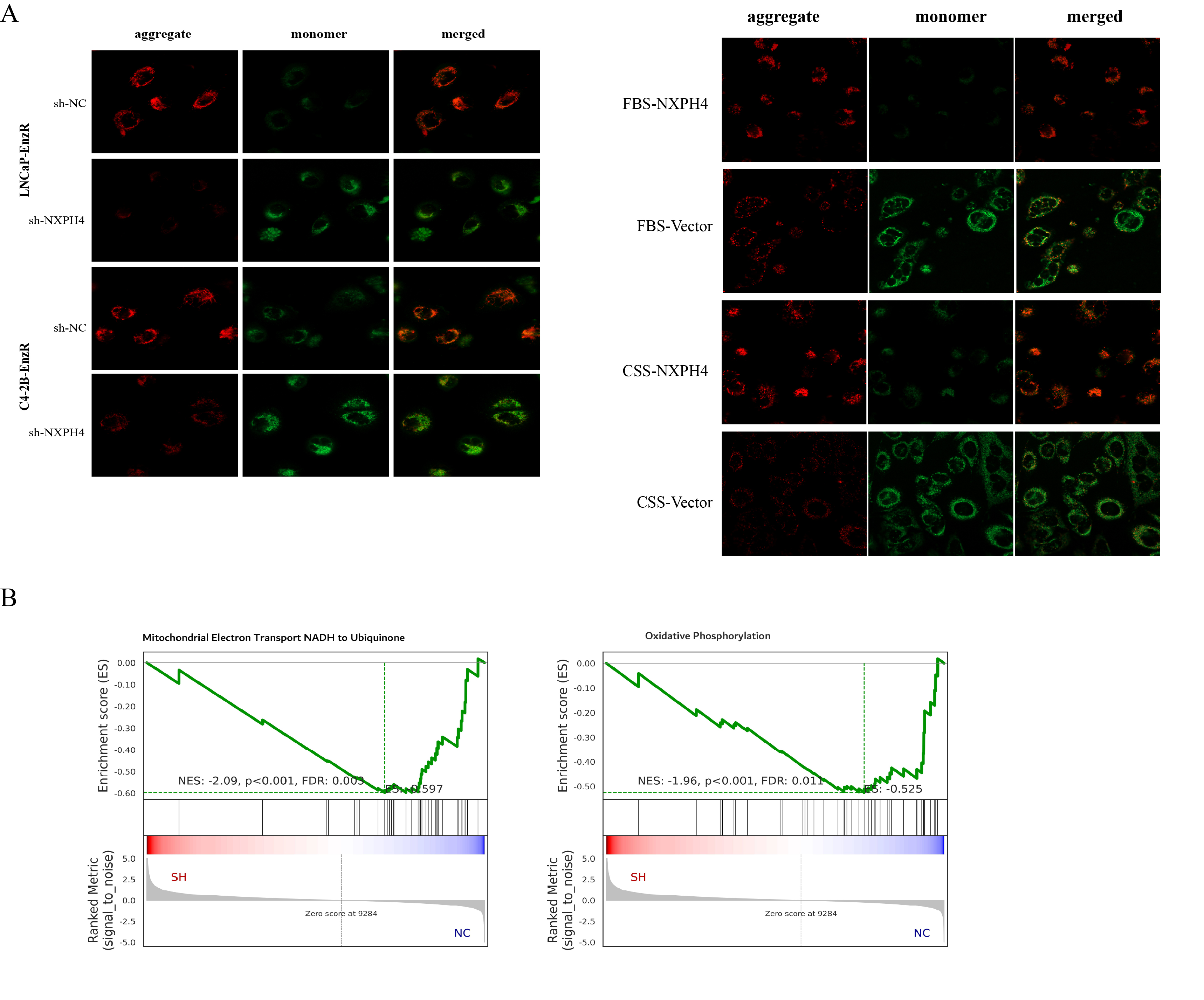

Supplement: Supplementary file 9 — Supplementary Figure 3 [file 41420_2026_2944_MOESM9_ESM.jpg]

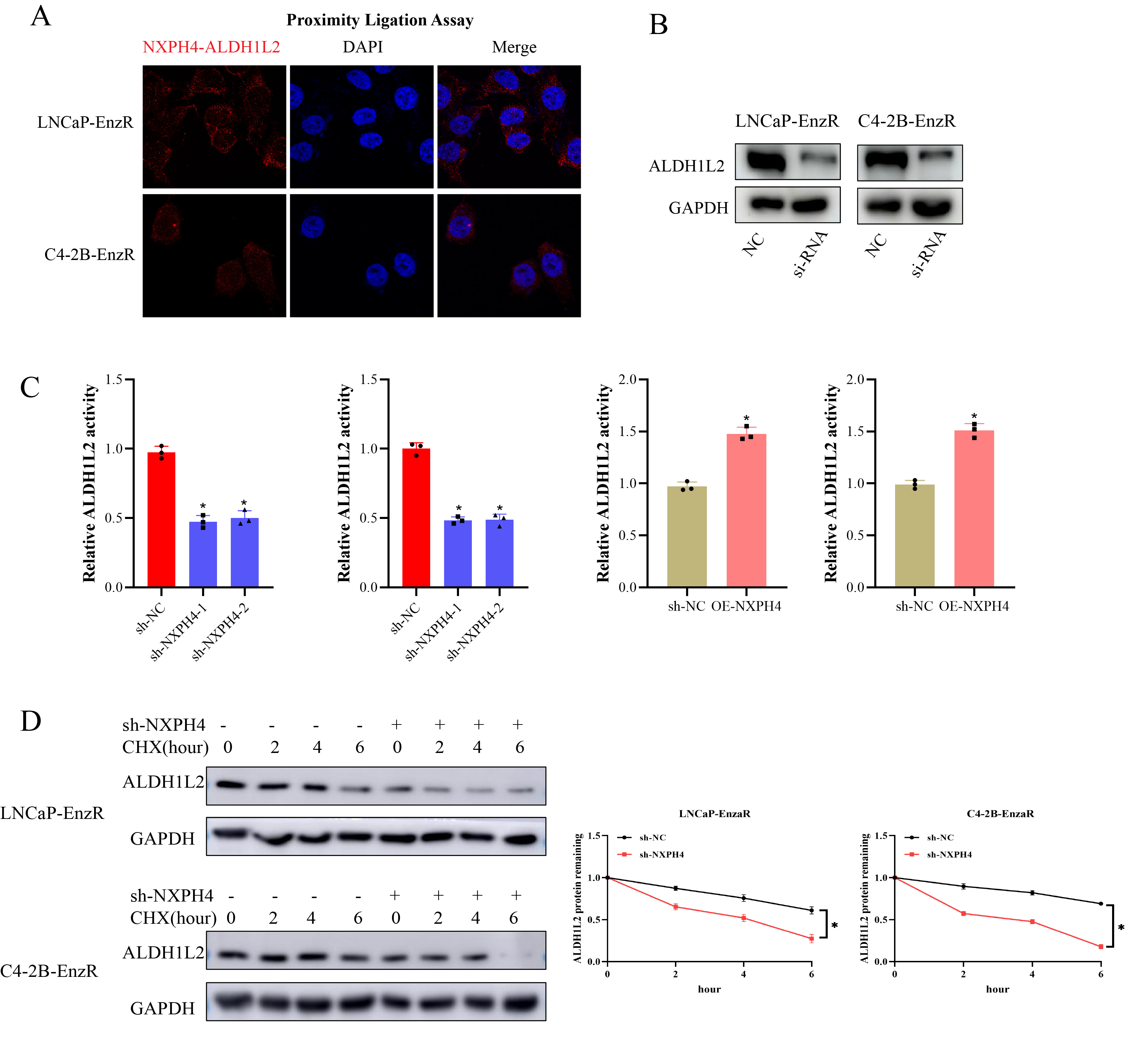

Supplement: Supplementary file 10 — Supplementary Figure 4 [file 41420_2026_2944_MOESM10_ESM.jpg]

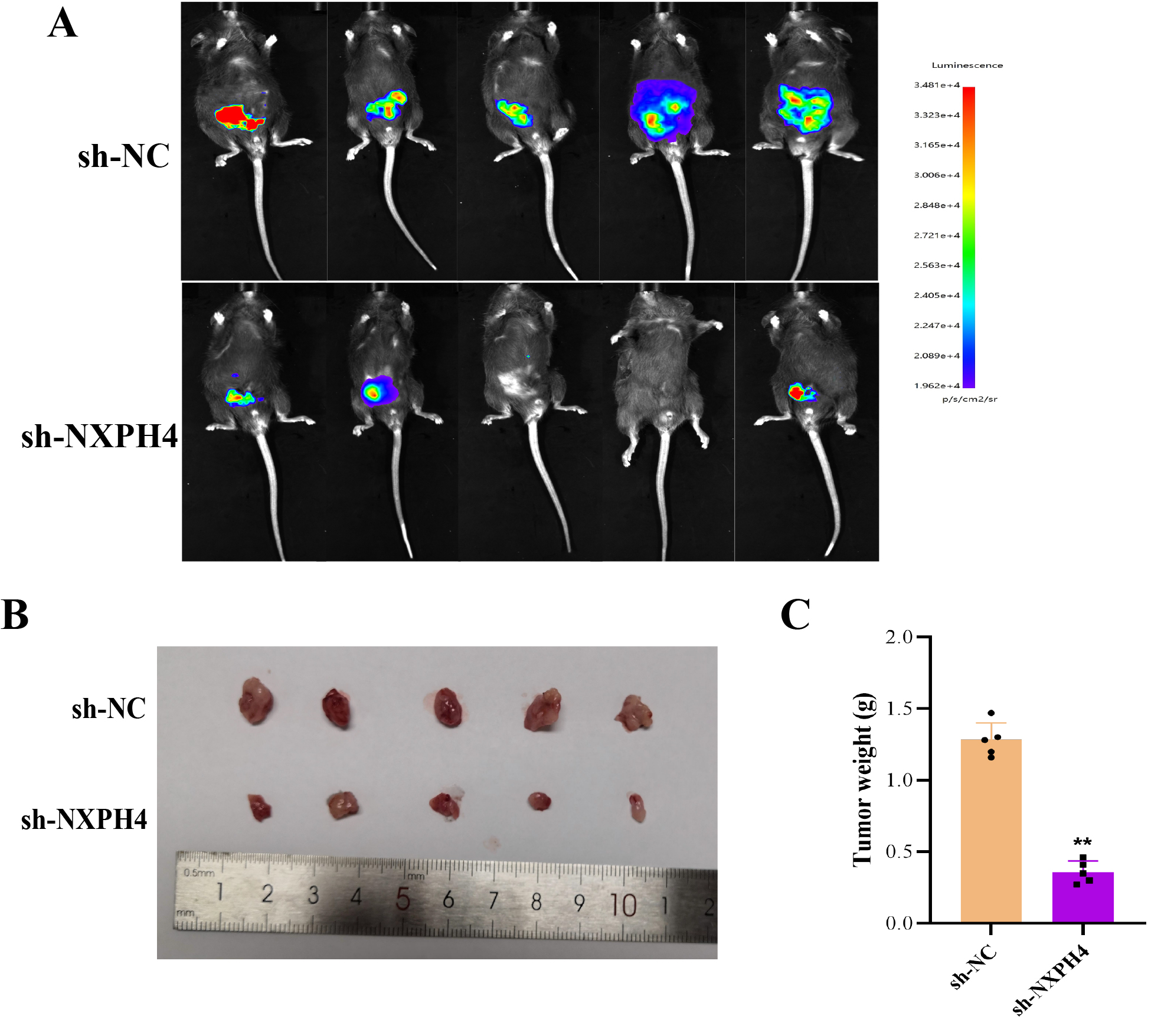

Supplement: Supplementary file 11 — Supplementary Figure 5 [file 41420_2026_2944_MOESM11_ESM.jpg]
